# KLF5 Promotes Tumor Progression and Parp Inhibitor Resistance in Ovarian Cancer

**DOI:** 10.1002/advs.202304638

**Published:** 2023-09-13

**Authors:** Yong Wu, Siyu Chen, Yang Shao, Ying Su, Qin Li, Jiangchun Wu, Jun Zhu, Hao Wen, Yan Huang, Zhong Zheng, Xiaojun Chen, Xingzhu Ju, Shenglin Huang, Xiaohua Wu, Zhixiang Hu

**Affiliations:** ^1^ Department of Gynecologic Oncology Fudan University Shanghai Cancer Center Shanghai Key Laboratory of Medical Epigenetics International Co‐laboratory of Medical Epigenetics and Metabolism Institutes of Biomedical Sciences Shanghai Medical College Fudan University Shanghai 200032 China; ^2^ Department of Oncology Shanghai Medical College Fudan University Shanghai China

**Keywords:** homologous recombination repair, *KLF5*, ovarian cancer, PARPi resistance, *RAD51*, super‐enhancer

## Abstract

One major characteristic of tumor cells is the aberrant activation of epigenetic regulatory elements, which remodel the tumor transcriptome and ultimately promote cancer progression and drug resistance. However, the oncogenic functions and mechanisms of ovarian cancer (OC) remain elusive. Here, super‐enhancer (SE) regulatory elements that are aberrantly activated in OC are identified and it is found that SEs drive the relative specific expression of the transcription factor *KLF5* in OC patients and poly(ADP‐ribose) polymerase inhibitor (PARPi)‐resistant patients. *KLF5* expression is associated with poor outcomes in OC patients and can drive tumor progression in vitro and in vivo. Mechanistically, *KLF5* forms a transcriptional complex with *EHF* and *ELF3* and binds to the promoter region of *RAD51* to enhance its transcription, strengthening the homologous recombination repair (HRR) pathway. Notably, the combination of suberoylanilide hydroxamic acid (SAHA) and olaparib significantly inhibits tumor growth and metastasis of PARPi‐resistant OC cells with high *KLF5*. In conclusion, it is discovered that SEs‐driven *KLF5* is a key regulatory factor in OC progression and PARPi resistance; and potential therapeutic strategies for OC patients with PARPi resistance and high *KLF5* are identified.

## Graphical abstract

This study depicts the landscape of abnormally activated super‐enhancer regulatory elements in ovarian cancer (OC) and identifies two super‐enhancers that regulate Kruppel‐like factor 5 (*KLF5*) transcription, resulting in *KLF5* self‐transcriptional regulation. *KLF5* forms a transcription complex with *EHF* and *ELF3*, remodels *RAD51* transcription, strengthens the homologous recombination repair pathway in OC cells, and ultimately promotes OC progression and PARPi resistance.

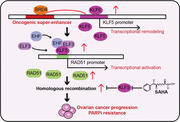

## 1 Introduction

Ovarian cancer (OC) is a malignant tumor affecting the reproductive system in women, with a notable high incidence rate. Among gynecological tumors, OC exhibits one of the highest mortality rates.^[^ ^1^ ^]^ Epithelial ovarian cancer (EOC) constitutes ≈90% of OC cases, encompassing subtypes such as high‐grade serous ovarian cancer (HGSOC, ≈80%), low‐grade serous ovarian carcinoma (LGSOC), ovarian endometrioid carcinoma (OEC), ovarian clear cell carcinoma (OCCC), and mucinous ovarian cancer (MOC).^[^ ^2^ ^]^ Except for frequent TP53 mutations and a few infrequent but important recurrent somatic mutations in protein‐coding genes such as *BRCA1/2*, *ARID1A*, and *CDK2*, the overall mutation rate in OC is very low, making it extremely challenging to develop drugs targeting protein‐coding gene mutations.^[^ ^3^ ^]^ The standard first‐line treatment for OC involves a combination of surgery and platinum‐based chemotherapy. However, nearly 70% of patients with late‐stage OC develop resistance to platinum‐based drugs, leading to tumor recurrence and significantly reduced survival time.^[^ ^4^ ^]^ Hence, there is an urgent need for research in other areas, such as exploring the non‐coding regions of the genome and the tumor transcriptome, to comprehend the underlying mechanisms of OC progression and develop novel treatment strategies.

The diversity and complexity of the tumor transcriptome are major features of cancer. Increasing evidence suggests that epigenetic regulatory elements, such as promoters, enhancers, and super‐enhancers (SEs), appear specifically or abnormally activated in tumors, promoting tumorigenesis, metastasis, and drug resistance by remodeling the tumor transcriptome to generate tumor‐addictive or tumor‐specific transcripts (TSTs).^[^ ^5^, ^6^, ^7^, ^8^ ^]^ This is more prevalent in tumors with low mutation rates, such as ovarian and liver cancers.^[^ ^9^, ^10^, ^11^, ^12^ ^]^ Enhancers are non‐coding regions of the genome that contain cis‐regulatory elements and promote transcription of target genes. SEs are large clusters of enhancers associated with high histone modifications (H3K4me1, H3K27Ac, etc.) and core factors such as *BRD4*.^[^ ^13^ ^]^ Compared to enhancers, SEs have stronger transcriptional activation ability and powerful transcriptional regulation function, precisely controlling the transcriptional level of cells and maintaining their specific states. In tumors, abnormal activation of SEs promotes initiation, progression, and drug resistance.^[^ ^14^ ^]^ In triple‐negative breast cancer, abnormal activation of SEs results in the transcription of a tumor‐specific transcript, *MARCO‐TST*, which encodes a protein that binds to PLOD2, enhances HIF‐1α protein stability and promotes proliferation and metastasis of triple‐negative breast cancer.^[^ ^15^ ^]^ Abnormal activation of SEs also directly regulates the transcription of oncogenes and triggers ovarian tumor development and malignancy.^[^ ^10^, ^16^ ^]^

In recent years, the development of poly (ADP‐ribose) polymerase (PARP) inhibitors (PARPi) has shown a powerful effect in treating patients.^[^ ^17^, ^18^, ^19^ ^]^ PARP is a key enzyme involved in the repair of single‐stranded DNA breaks (SSBs) in eukaryotic cells.^[^ ^20^ ^]^ Inhibition of PARP leads to the accumulation of SSBs, promoting DNA double‐strand breaks (DSBs). The repair process of DSBs mainly depends on homologous recombination repair (HRR) and tumor suppressor genes such as *BRCA1/2*. Therefore, inhibition of PARP in these cells can accumulate excessive DSBs, producing synergistic lethal effects (synthetic lethality) and ultimately inhibiting OC growth and malignant progression.^[^ ^21^ ^]^ Drug resistance is a common problem in OC patients treated with PARPi. Meanwhile, homologous recombination (HR)‐proficient cells are relatively insensitive to PARPi. Whether OC controls transcriptome remodeling and regulates PARPi resistance through abnormal activation of epigenetic regulatory elements has not been systematically reported.

*KLF5* (Kruppel‐like factor 5) is a member of the Kruppel‐like factor family, which acts as a transcription factor. *KLF5* is specifically expressed in various cells and plays an important role in development, metabolism, and cellular pluripotency.^[^ ^22^ ^]^ *KLF5* drives the progression and metastasis of various tumors.^[^ ^23^ ^]^ In esophageal squamous cell carcinoma, *KLF5* binds to *SOX2*, activates *STAT3* transcription, and promotes esophageal squamous cell carcinoma (ESCC) development.^[^ ^24^ ^]^ *KLF5* can also form a complex with TP63, YAP1, and CBP/EP300, which activates the transcription of related oncogenes by forming 3D chromatin loops and promoting epithelioid tumor progression.^[^ ^25^ ^]^ Notably, several studies have highlighted the regulatory relationship between *KLF5* and SEs, in which *KLF5* is associated with other transcription factors to form interconnected circuitry and established SE‐regulated circuits to remodel oncogene transcription and drive cancer progression.^[^ ^26^, ^27^ ^]^ However, the characteristics, function, and involvement of *KLF5* in mediating PARPi resistance in OC remain unclear.

This study mapped SEs elements to identify abnormally activated SEs in OC and downstream core regulatory genes. Unexpectedly, we found that SEs drive relatively specific high expression of *KLF5* in OC. High *KLF5* expression promotes *RAD51* transcriptional remodeling, enhancing HRR pathways and PARPi resistance in OC. Targeted inhibition of *KLF5* enhanced the sensitivity of OC cells to olaparib, suggesting that *KLF5* may be a core therapeutic target for OC progression and PARPi resistance.

## 2 Results

### 2.1 The Landscape of Aberrantly Activated SEs in OC

**Figure 1:**
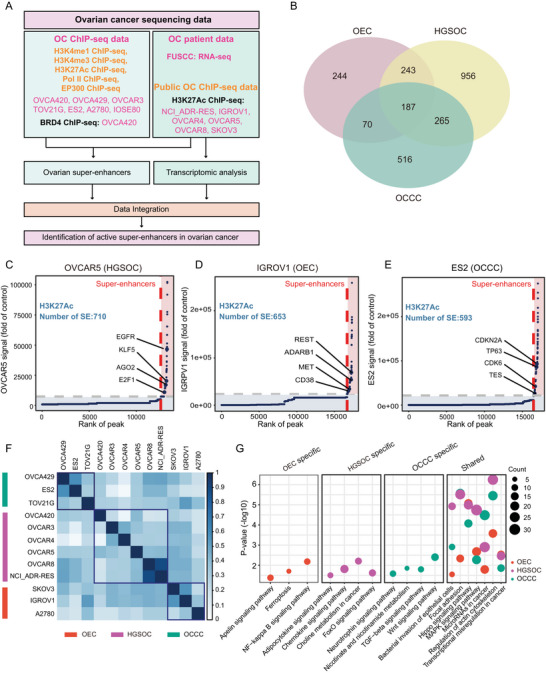
Depicting super‐enhancer elements in OC. A) Flowchart of the analysis strategy used to identify abnormally activated SEs in OC. ChIP‐seq of H3K4me1, H3K4me3, H3K27Ac, polII, and EP300 in six OC cells (OVCA420, OVCA429, OVCAR3, TOV21G, ES2, and A2780) and one normal ovarian epithelial cell (IOSE80) were performed. The public H3K27Ac ChIP‐seq data of NCI_ADR‐RES, IGRPV1, OVCAR4, OVCAR5, OVCAR8, and SKOV3, were collected, and RNA‐seq data, including eight normal ovarian tissues and 30 OC tissues from FUSCC was collected to perform transcriptional analysis. Data were integrated to identify active SEs and related downstream targeted genes. B) The venn diagram shows the number of SEs in each subtype of OC. 187 SEs were shared in three types of OC. C–E) The number of SEs in OVCAR5 (C; SE = 710), IGRPV1 (D; SE = 653), and ES2 (E; SE = 593) cells and the representation of adjacent representative genes. SEs are marked with black dots. F) The matrix shows pair‐wise similarity of SEs detected in different cell types. The degree of similarity is colored in proportion to the overlap percentage. The top part (green rectangle) represents the endome type of OC cell lines, the middle part (pink rectangle) represents the high‐grade type of OC cell lines, and the bottom part (orange rectangle) represents the OCCC type of OC cell lines. G) The specific and shared KEGG signaling pathways of SEs adjacent gene enrichment in three OC subtypes.

To clarify the characteristics of aberrantly activated SEs elements in OC, we performed chromatin immunoprecipitation sequencing (ChIP‐seq) of H3K4me1, H3K4me3, H3K27Ac, polII, and EP300 in six OC cells (OVCA420, OVCA429, OVCAR3, TOV21G, ES2, and A2780) and one normal ovarian epithelial cell (IOSE80), and *BRD4* ChIP‐seq sequencing of OVCA420 cells. Next, we conducted RNA‐seq data analysis of OC from the Fudan University Shanghai Cancer Center (FUSCC) and collected publicly available H3K27Ac ChIP‐seq data for six ovarian cancer cell lines (NCI_ADR‐RES, IGROV1, OVCAR4, OVCAR5, OVCAR8, and SKOV3) from the ENCODE website (https://www.encodeproject.org/). We integrated and analyzed the data to identify SEs elements that were not present in normal ovarian cells but aberrantly activated in OC, as well as potential downstream gene networks that may be regulated by these elements (**Figure** **1**A). We focused on regions that were enriched with H3K27Ac signals and located far from the transcription start site (TSS) of genes (2.5 kb upstream/downstream of TSS), which are enhancer regions in previous studies.^[^ ^28^ ^]^ We used ROSE software (https://hpc.nih.gov/apps/ROSE.html) to analyze the H3K27Ac data of 12 OC cell lines and obtained information on the SEs elements. Notably, we identified 2481 aberrantly activated SEs elements in OC, including 1651 in HGSOC, 1038 in OCCC, and 744 in OEC (Figure 1B; Table S1, Supporting Information). One hundred eighty‐seven super‐enhancer elements were present in all three types of OC. SEs were mainly located in the intergenic and intronic regions, a common distribution pattern in other tumors^[^ ^28^ ^]^ (Figure S1, Supporting Information). We further observed that these SEs elements exhibited H3K27Ac and H3K4me1 signals, consistent with the previously reported characteristics of SEs elements in other tumors.^[^ ^26^ ^]^ We selected representative cell lines of the three OC subtypes to investigate adjacent genes that may be regulated by SEs. For example, *EGFR* and *KLF5* were enriched in HGSOC cells (Figure 1C); *REST* and *MET* were enriched in OEC cells (Figure 1D); and *CDKN2A* and *CDK6* were enriched in OCCC cells (Figure 1E). We also compared the extent to which SEs appeared in the three cell line subtypes and found that the cell lines belonging to the same subtype had a higher degree of SE similarity (Figure 1F). To uncover the key signaling pathways related to SEs in different subtypes of OC, we analyzed adjacent genes enriched by SEs and found that the OEC subtype was enriched in ferroptosis, NF‐kappa B, and other signaling pathways; the HGSOC subtype was enriched in chemotaxis and choline metabolism signaling pathways; and the OCCC subtype was mainly enriched in TGF‐β and Wnt signaling pathways. All three subtypes were enriched in the Hippo, MAPK, and transcriptional abnormalities signaling pathways (Figure 1G). Collectively, our results provide a comprehensive understanding of aberrantly activated SEs in OC as well as their presence and characteristics in different OC subtypes.

### 2.2 SEs Drive *KLF5* Transcription and *KLF5* Self‐Transcription Regulation

**Figure 2:**
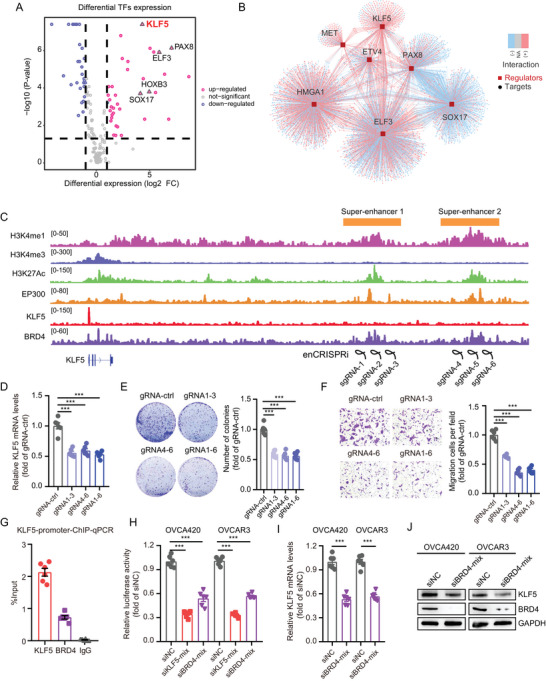
SEs drive *KLF5* transcription and self‐transcriptional regulation of *KLF5* in OC. A) The volcano plot shows differential expression of TFs in OC using FUSCC cohort RNA‐seq data, including eight normal ovarian tissues and 30 OC tissues. The expression of *KLF5* was abnormally high in OC with the most significant statistical difference (P = 4.09E‐08; Fold‐change = 21.257). B) Differential expression of TFs and regulatory networks of its downstream genes. C) ChIP‐seq of H3K4me1, H3K4me3, H3K27Ac, EP300, *KLF5* and *BRD4* at *KLF5* promoter and two upstream SEs. Guide RNAs were designed to target inhibition the activity of SEs of *KLF5* using the enCRISPRi system. D) The mRNA levels of *KLF5* in OVCA420 cells infected with dCas9‐KRAB, sgRNAs targeting *KLF5* SEs, and MCP‐LSD1 lentivirus. E,F) Blockade the *KLF5* SEs inhibits colony number formation (E) and migration ability (F) in OVCA420 cells using enCRISPRi lentivirus. G) The ChIP‐qPCR assay shows the binding of *KLF5* and *BRD4* at the *KLF5* promoter in the OVCA420 cell. H) The luciferase activity of *KLF5* promoter transfected with *KLF5* or *BRD4* siRNAs in OVCA420 and OVCAR3 cells. I,J) The mRNA levels (I) and protein levels (J) of *KLF5* transfected with *BRD4* siRNAs in OVCA420 and OVCAR3 cells. Values represent the mean ± SEM, *n* = 6 in (D–I). ^***^ *p* < 0.001.

Since transcription factors (TFs) participate in the regulatory network of SEs, we analyzed differentially expressed TFs in 30 OC and eight normal ovarian RNA sequencing data from FUSCC, in which aberrantly activated super‐enhancer elements were found near the genes of these TFs. A volcano plot suggested that *KLF5*, *PAX8*, *SOX17*, *ELF3*, and *HOXB3* are highly expressed in OC tissues (**Figure** **2**A; Table S2, Supporting Information). Furthermore, we constructed a regulatory network of these TFs for potential downstream target genes (Figure 2B). Unexpectedly, we found activation of two super‐enhancer regulatory elements upstream of the *KLF5* promoter, which was further observed in OVCA420 and OVCAR5 OC cells (Figure 2C; Figure S2A, Supporting Information). In OVCA420 cells, the two SEs showed histone modification signals of H3K4me1 and H3K27Ac, with binding of EP300 and *BRD4*, accompanied by low modification signal levels of H3K4me3. We also observed that *KLF5* and *BRD4* protein binding occurs in their promoter regions, suggesting that *KLF5* may be subjected to SEs transcriptional regulation and self‐regulation. To test this hypothesis, we first used the enCRISPRi system to inhibit the activity of these two super‐enhancers and found that inhibition of the two SEs activities, alone or together, significantly dampened the H3K27Ac levels of SEs (Figure S2B, Supporting Information) and downregulated *KLF5* mRNA levels (Figure 2D). Consistently, inhibition of the two SEs activities abolished the colony formation (Figure 2E) and invasion abilities (Figure 2F) of OVCA420 cells. Furthermore, we confirmed the binding of *KLF5* and *BRD4* in *KLF5* promoter regions using ChIP‐qPCR assay (Figure 2G) and designed small interfering RNA (siRNA) targeting *KLF5* and *BRD4* in OVCA420 cells. Knockdown of *KLF5* or *BRD4* significantly inhibited *KLF5* promoter activity (Figure 2H). Moreover, *BRD4* knockdown significantly decreased *KLF5* mRNA (Figure 2I) and protein levels (Figure 2J). Together, these results support our hypothesis that abnormal activation of SEs regulates the transcription of *KLF5*, and *KLF5* further binds to its own promoter region to maintain self‐high transcriptional expression.

### 2.3 High Expression of *KLF5* is Specific to OC and Associated with Poor Prognosis

**Figure 3:**
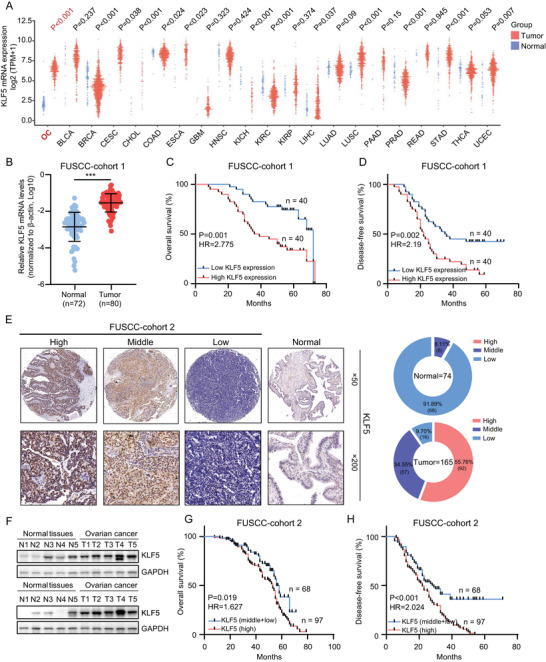
*KLF5* is specifically overexpressed in OC and clinically associated with patient prognosis. A) The mRNA levels of *KLF5* in different normal and tumor samples in the TCGA dataset. B) The mRNA levels of *KLF5* in normal and OC samples in FUSCC cohort 1. C,D) Kaplan–Meier curves of overall survival (C) and disease‐free survival (D) in FUSCC cohort 1 stratified by *KLF5* mRNA levels of OC tissues. E) Representative immunostaining images of *KLF5* in 74 normal and 165 OC tissues of FUSCC cohort 2. F) Immunoblotting for *KLF5* protein levels in normal and OC tissues. G,H) Kaplan–Meier curves of overall survival (G) and disease‐free survival (H) in FUSCC cohort 2 stratified by *KLF5* protein levels of OC tissues. ^***^ *p* < 0.001.

To elucidate the characteristics of *KLF5*, we collected RNA‐seq data from 21 types of normal and cancer tissues from The Cancer Genome Atlas (TCGA) public database (https://cancergenome.nih.gov/). We found that *KLF5* was highly expressed in tumors such as OC, bile duct, and gastric cancer. Among them, the difference in *KLF5* expression was particularly significant in OC (**Figure** **3**A; *p* < 0.001). The RNA‐seq data from FUSCC in Figure 2A also highlighted the overexpression of *KLF5* (fold change = 21.257, *p* = 4.09E‐08), indicating that *KLF5* may be a relatively specific driver in OC. Further validation was conducted in FUSCC cohort 1, which included 72 cases of normal ovarian tissues and 80 cases of OC tissues. A similar high expression pattern of *KLF5* was found in OC tissues of FUSCC cohort 1 (Figure 3B; *p* < 0.001), and OC patients with high *KLF5* expression had lower overall survival rates (Figure 3C; *p* = 0.001, hazard ratio = 2.775) and disease‐free survival rates (Figure 3D; Table S3, Supporting Information; *p* = 0.002, hazard ratio = 2.19). Accordingly, we validated *KLF5* protein levels in FUSCC cohort 2 (including 74 cases of normal ovarian tissue and 165 cases of OC tissue). We found that the protein level of *KLF5* was also highly expressed in OC tissues (Figure 3E,F). OC patients with high *KLF5* protein levels also had lower overall survival (Figure 3G; *p* = 0.019, hazard ratio = 1.627) and disease‐free survival (Figure 3H; Table S4, Supporting Information; *p* < 0.001, hazard ratio = 2.024) rates. Univariate analysis revealed that *KLF5* protein levels were related to the tumor residual margin (*p* = 0.031) and diaphragm metastasis (Figure S3A, Supporting Information; *p* = 0.038), and multivariate analysis confirmed a correlation between *KLF5* expression and tumor residual margins (Figure S3B, Supporting Information; *p* = 0.014). These results indicate that *KLF5* is abnormally highly expressed in OC and is associated with a poor prognosis. *KLF5* may be a relatively specific driver of OC development.

### 2.4 *KLF5* Drives the Growth and Metastasis of OC

**Figure 4:**
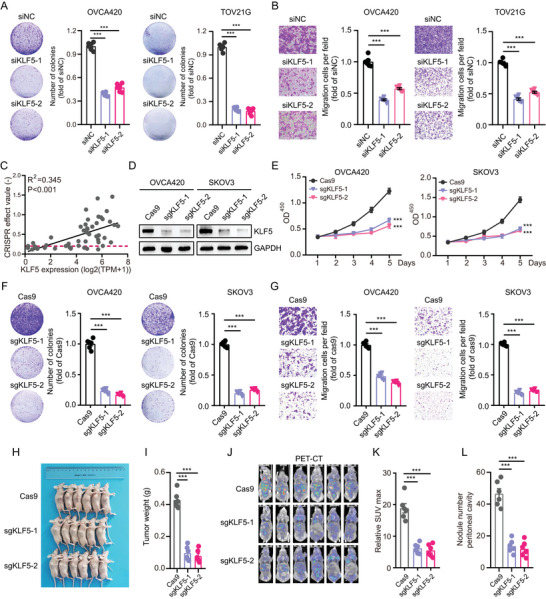
*KLF5* drives OC tumor growth and metastasis. A,B) Colony formation assays (A) and transwell migration assays (B) of OVCA420 and TOV21G cells transfected with *KLF5* siRNAs or control siRNA. C) The correlation between *KLF5* mRNA levels with CRISPR affected value (proliferative ability) in OC cell lines from the Depmap dataset. D) Immunoblotting for *KLF5* protein levels in OVCA420 and SKOV3 cells infected with Cas9 and *KLF5* sgRNAs. E) Cell Counting Kit‐8 (CCK‐8) assay of OVCA420 and SKOV3 cells infected with Cas9 and *KLF5* sgRNAs. F,G) Colony formation assays (F) and transwell migration assays (G) of OVCA420 and SKOV3 cells infected with Cas9 and *KLF5* sgRNAs. H) Xenograft tumors in nude mice. SKOV3 cells were infected with Cas9 and control gRNA or sg*KLF5*‐1 or sg*KLF5*‐2 knockdown lentivirus and subcutaneously injected into the flanks of 6 weeks old nude mice. I) Knockout of *KLF5* reduced the weight of xenograft tumors (*n* = 6 mice per group). J) PET‐CT shows the abdominal tumor metastasis of TOV21G cells infected with Cas9 and *KLF5* sgRNAs. K,L) The relative SUV max and nodule number peritoneal cavity of TOV21G cells infected with Cas9 and *KLF5* sgRNAs. Values represent the mean ± SEM, *n* = 6 in (A,B; F–L). ^***^ *p* < 0.001.

Because of its high expression of *KLF5* in OC, we explored the oncogenic function of *KLF5* in OC. We designed two siRNAs targeting *KLF5* and found that the knockdown of *KLF5* significantly inhibited colony formation (**Figure** **4**A) and migration (Figure 4B) in OVCA420 and TOV21G OC cells. Clustered Regularly Interspaced Short Palindromic Repeats/Cas9 (CRISPR/Cas9) has strong gene editing ability and is widely used for gene functional screening. We analyzed the expression and CRISPR/Cas9 proliferative screening data of *KLF5* in 49 OC cells in the Depmap database (https://depmap.org/portal/). Notably, there was a significant positive correlation between high *KLF5* expression and cell proliferation, suggesting a crucial role of *KLF5* in promoting the OC cell proliferation process (Figure 4C; R square = 0.345, *p* < 0.001). We then constructed two guide RNA plasmids targeting *KLF5*, packaged them with the CRISPR plasmid into lentivirus, and infected OVCA420 and SKOV3 cells with high expression of *KLF5*. Western blot analysis showed an efficient knockdown of these OC cells (Figure 4D). CCK‐8, colony formation, and transwell assays showed that *KLF5* knockdown significantly inhibited proliferation (Figure 4E,F) and migration (Figure 4G) of OC cells with high *KLF5*. To validate our in vivo findings, SKOV3 cells were infected with Cas9 and control gRNA or sg*KLF5* knockdown lentivirus and subcutaneously injected into the flanks of 6‐week‐old nude mice. The results showed that *KLF5* knockdown significantly reduced the tumor size of OC cells (Figure 4H,I). TOV21G cells were infected with Cas9 and control gRNA or sg*KLF5* knockdown lentivirus and intraperitoneally injected into nude mice. PET‐CT showed that *KLF5* knockdown in OC cells reduced SUV uptake (Figure 4J,K) and the number of abdominal tumor nodules (Figure 4L). We also constructed *KLF5* overexpression lentiviruses to infect Hey and OVCAR8 cells with low *KLF5* expression levels (Figure S4A, Supporting Information). Our data suggested that *KLF5* overexpression enhanced the colony formation ability of Hey and OVCAR8 cells (Figure S4B, Supporting Information) and migration ability (Figure S4C, Supporting Information) in vitro. Hey cells were further infected with PCDH‐3XFlag‐control or PCDH‐3XFlag‐*KLF5* lentivirus and subcutaneously injected into the flanks of 6‐week‐old nude mice. Overexpression of *KLF5* promoted OC tumor formation in vivo (Figure S4D,E, Supporting Information). Collectively, these results indicated that *KLF5* promotes OC growth and metastasis.

### 2.5 *KLF5* Regulates *RAD51* Transcription and HRR Pathway

**Figure 5:**
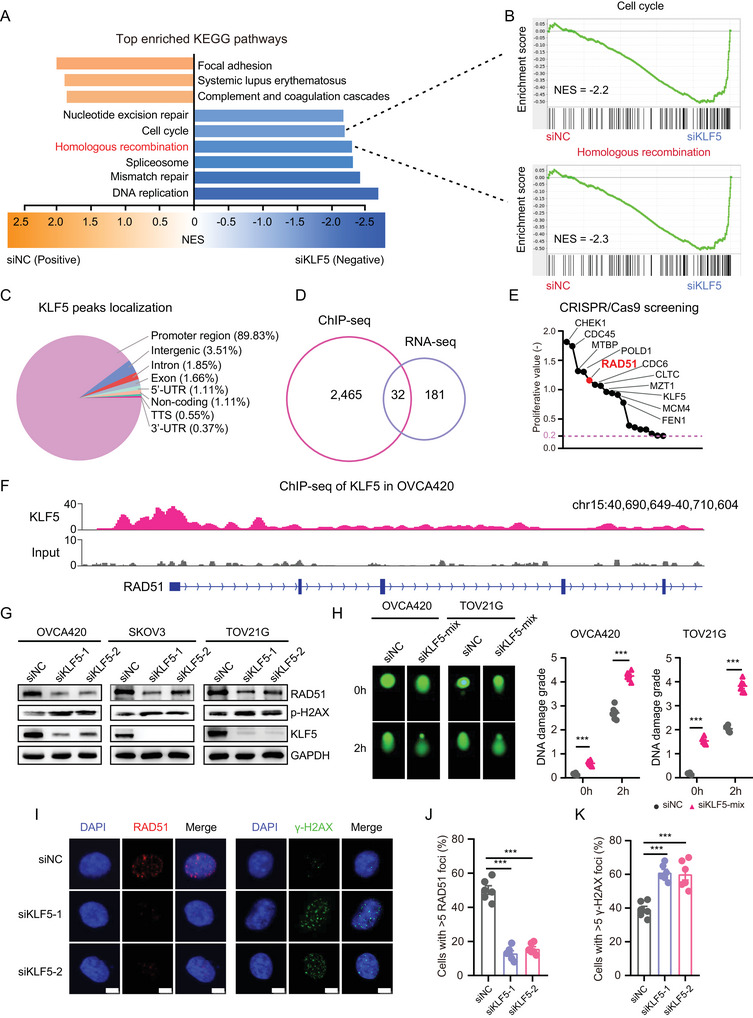
*KLF5* regulates *RAD51* transcription and HRR pathway. A) Top six enriched KEGG downregulated pathways and three upregulated pathways in OVCA420 cells transfected with *KLF5* siRNAs or control siRNA. B) GSEA enriched cell cycle (NES = −2.2) and homologous recombination (NES = −2.3) pathway in OVCA420 cell transfected with *KLF5* siRNAs or control siRNA. C) The genome‐wide binding sites of *KLF5* in OVCA420 cells, most of which were located in promoter regions. D) Venn diagram of *KLF5* ChIP‐seq (fold‐enrichment with input > = 5) and RNA‐seq data (fold‐change with siNC < = 0.5) shows 32 genes transcriptionally regulated by *KLF5* in OVCA420 cells. E) The proliferative value of *KLF5* target genes in OVCA420 from the Depmap dataset suggests these genes participated in OC cell proliferation. F) The ChIP‐seq data of *KLF5* in OVCA420 shows the binding of *KLF5* at *RAD51* promoter regions. G) Immunoblotting for *RAD51*, p‐H2AX, *KLF5* protein levels in OVCA420, SKOV3, and TOV21G cells transfected with *KLF5* siRNAs or control siRNA. H) Comet assay in OVCA420 and TOV21G cells transfected with *KLF5* siRNAs or control siRNA. I) Immunofluorescence staining with *RAD51* antibody and γ‐H2AX antibody in OVCA420 cells treated with cisplatin (10 µm, 1 h). Cell nuclei were counterstained with DAPI. J,K) Quantitative results of *RAD51* foci (J) and γ‐H2AX foci (K). Cells with more than five foci were defined as positive and counted six times (at least 50 cells per cell line each time). Values represent the mean ± SEM, *n* = 6 in (H, J,K). Scale bar = 10 µm. ^***^ *p* < 0.001.

To further investigate the molecular mechanism by which *KLF5* promotes OC progression, we analyzed the RNA‐seq gene expression profiles of control and *KLF5* knockdown OVCA420 cells (fold change ≤ 0.5). GSEA showed that the top five downregulated signaling pathways in *KLF5* knockdown cells were enriched in DNA replication, mismatch repair, spliceosome, HR, and the cell cycle (**Figure** **5**A,B). Since *KLF5* acts as a transcription factor to perform biological functions by controlling binding to specific genome regions and regulating target gene transcription, we performed a ChIP‐seq of *KLF5* in OVCA420 cells. More than five times the enrichment difference was selected as *KLF5*‐specific binding sites controlled by input. The results suggested that 2497 *KLF5* binding sites were obtained, mainly located in the gene promoter region (Figure 5C,D; Table S5, Supporting Information). *KLF5* binding sites were also enriched with H3K4me3 and H3K27Ac histone modifications, representing active promoter signatures (Figure S5A, Supporting Information). Next, we analyzed the ChIP‐seq and RNA‐seq data (Table S6, Supporting Information) and found that 32 target genes might be transcriptionally regulated by *KLF5* (Figure 5D). Using CRISPR/Cas9 function screening data of OVCA420 cells, we noticed that 18 of the 32 genes were related to cell proliferation (Figure 5E; proliferative cut‐off value ≤ −0.2).

Recent studies have confirmed that the HRR pathway, including its core‐related genes *RAD51*, is crucial in promoting cancer progression.^[^ ^29^ ^]^ More importantly, HRR closely regulates PARPi resistance in OC.^[^ ^30^ ^]^ *RAD51* participates in DNA HRR by forming a complex with *BRCA2* and other proteins and binding to the broken DNA region.^[^ ^31^ ^]^ It is abnormally highly expressed in various tumors, including OC (GEPIA, http://gepia.cancer‐pku.cn). *RAD51* overexpression promotes cancer progression.^[^ ^32^ ^]^ Moreover, tumor cells with high *RAD51* expression exhibit PARPi resistance.^[^ ^33^, ^34^ ^]^ As the knockdown of *KLF5* significantly inhibited the HRR pathway in OC OVCA420 cells, and functional sequencing and analysis of these 18 genes revealed that they contained the core homologous recombination gene *RAD51*, we speculated that *KLF5* might remodel *RAD51* transcription. To test this hypothesis, we first observed *KLF5* binding sites in *RAD51* genomic regions and found significant enrichment in the *RAD51* promoter (Figure 5F). Importantly, *KLF5* knockdown significantly reduced *RAD51* mRNA levels (Figure S5B, Supporting Information) and protein levels (Figure 5G) and increased the protein levels of phosphorylated γ‐H2AX in OC cells. In addition, *KLF5* knockdown promoted DNA damage in OVCA420 and TOV21G OC cells (Figure 5H). Immunofluorescence staining revealed that inhibition of *KLF5* reduced *RAD51* foci and increased γ‐H2AX foci (Figure 5I–K), suggesting that *KLF5* plays a crucial role in regulating HRR in ovarian cancer cells. Furthermore, analysis of *KLF5* binding peaks in the genome of HRR‐related genes showed that *KLF5* binds to the promoter regions of *CHEK1*, *RAD54L*, *EME1*, and *BLM* (Figure S5C, Supporting Information), and *KLF5* knockdown downregulated the mRNA levels of these HRR‐related genes (Figure S5D, Supporting Information), further supporting the important role of *KLF5* in the regulation of the HRR pathway. Taken together, these data suggested that *KLF5* could remodel *RAD51* transcription and regulate the HRR pathway in OC cells.

### 2.6 *KLF5* Forms a Transcription Complex with *EHF* and *ELF3*

**Figure 6:**
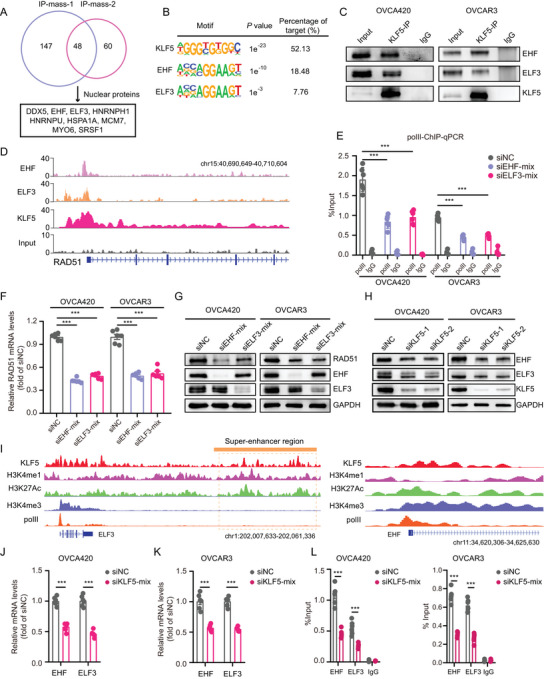
*KLF5* forms a transcriptional complex with *EHF* and *ELF3*. A) Immunoprecipitation‐mass spectrometry of OVCA420 cells immunoprecipitated with an anti‐*KLF5* antibody or control IgG and analyzed by SDS‐PAGE (10%). B) Motif analysis of *KLF5*, *EHF*, and *ELF3* in OVCA420 cells. C) Co‐immunoprecipitation analyses show the interaction between endogenous *KLF5*, *EHF* and *ELF3* in OVCA420 and OVCAR3 cells. D) The ChIP‐seq data shows the enrichment of *EHF*, *ELF3*, and *KLF5* in *RAD51* promoter region in OVCA420 cells. E) The enrichment ability of polII in *RAD51* promoter region in OVCA420 and OVCAR3 cells transfected with *EHF*, *ELF3* siRNAs, or control siRNA. F,G) The *RAD51* mRNA levels (F) and protein levels (G) in OVCA420 and OVCAR3 cells transfected with *EHF*, *ELF3* siRNAs, or control siRNA. H) Immunoblotting for *EHF*, *ELF3*, and *KLF5* protein levels in OVCA420 and OVCAR3 cells transfected with *KLF5* siRNAs or control siRNA. I) The ChIP‐seq data of *KLF5*, H3K4me1, H3K27Ac, H3K4me3, and polII shows the enrichment of *KLF5* in *ELF3* promoter and SE regions and *EHF* promoter region in OVCA420 cells. J,K) The *EHF* and *ELF3* mRNA levels in OVCA420 (J) and OVCAR3 (K) cells transfected with *KLF5* siRNAs or control siRNA. L) The enrichment ability of *EHF* and *ELF3* in *RAD51* promoter regions in OVCA420 cells and OVCAR3 cells transfected with *KLF5* siRNAs or control siRNA. Values represent the mean ± SEM, *n* = 6 in (E,F; J–L). ^***^ *p* < 0.001.

To better understand the specific transcriptional regulatory mechanism of *KLF5* on *RAD51*, we first performed protein immunoprecipitation followed by mass spectrometry (IP‐Mass) using a *KLF5*‐targeted antibody, and found 48 potential protein interactors with *KLF5*, including nine nuclear proteins (**Figure** **6**A). Binding motif analysis of the nine proteins, revealed that *EHF* and *ELF3* potentially overlap *KLF5* in the genome (Figure 6B). Consistently, the interaction between *KLF5*, *EHF*, and *ELF3* was readily detectable by co‐IP in OVCA420 and OVCAR3 cells (Figure 6C). Reverse co‐IP of *EHF* and *ELF3* also showed an association with *KLF5* in OVCA420 cells (Figure S6A, Supporting Information). To clarify the binding ability of *EHF* and *ELF3* to the *RAD51* promoter, we constructed PCDH‐3X‐Flag‐*EHF* and PCDH‐3X‐Flag‐*ELF3* overexpression plasmids, packaged them into lentivirus, and infected OVCA420 cells to perform ChIP‐seq. Our ChIP‐seq data showed that *EHF* and *ELF3* were also enriched in *RAD51* promoter regions, which were also co‐occupied with *KLF5* binding (Figure 6D). ChIP‐qPCR also revealed a significant enrichment of *EHF* and *ELF3* in *RAD51* promoter (Figure S6B, Supporting Information). Importantly, the knockdown of *EHF* and *ELF3* in OVCA420 and OVCAR3 OC cells weakened the binding of polII to *RAD51* promoter (Figure 6E) and reduced *RAD51* promoter activity (Figure S6C, Supporting Information), ultimately leading to the downregulation of *RAD51* mRNA (Figure 6F) and protein levels (Figure 6G). To further identify the key regulatory factors in this protein transcriptional complex, we transfected siRNA targeting *KLF5* into OVCA420 and OVCAR3 cells. We found that *KLF5* knockdown inhibited *EHF* and *ELF3* protein levels, indicating that *KLF5* may be the most crucial regulatory protein (Figure 6H). Furthermore, we observed that *KLF5* binds to *ELF3* promoter and upstream SE regions and EHF promoter regions (Figure 6I), suggesting that *KLF5* may also regulate the transcription of *EHF* and *ELF3*. Therefore, we transfected siRNA targeting *KLF5* into OVCA420 and OVCAR3 cells and found that *KLF5* knockdown also inhibited the mRNA levels of *EHF* and *ELF3* (Figure 6J,K) and the binding ability of *EHF* and *ELF3* to *RAD51* promoter (Figure 6L). Knockdown of *EHF* and *ELF3* decreased the mRNA levels of the HRR‐related genes (Figure S6D, Supporting Information) and inhibited colony formation (Figure S6E, Supporting Information) and migration (Figure S6F, Supporting Information) of OC cells in vitro. Together, these results indicate that *KLF5*, *EHF*, and *ELF3* form a transcriptional complex to remodel the transcriptional process of *RAD51* and control the HRR pathway in OC. *KLF5* may play a central role in this protein complex.

### 2.7 *EHF* and *ELF3* are Highly Expressed and Associated with Poor Prognosis

**Figure 7:**
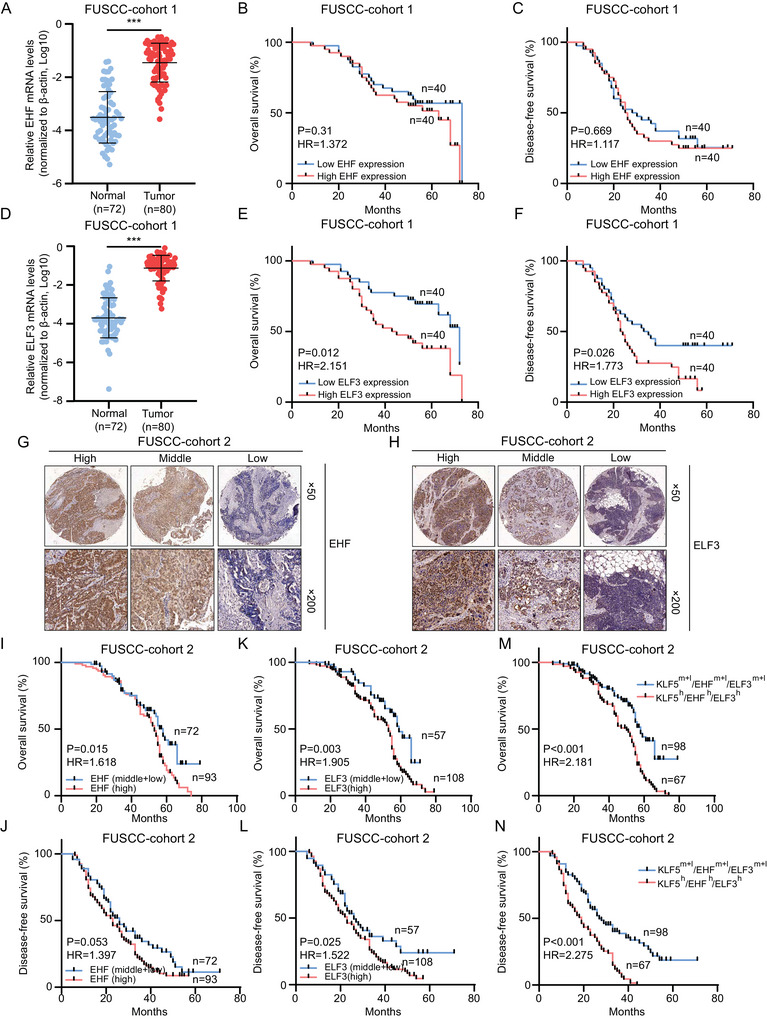
Clinical significance of *EHF* and *ELF3* in OC patients. A) The mRNA levels of *EHF* in normal and OC samples in FUSCC cohort 1. B,C) Kaplan–Meier curves of overall survival (B) and disease‐free survival (C) in FUSCC cohort 1 stratified by *EHF* mRNA levels of OC tissues. D) The mRNA levels of *ELF3* in normal and OC samples in FUSCC cohort 1. E,F) Kaplan–Meier curves of overall survival (E) and disease‐free survival (F) in FUSCC cohort 1 stratified by *ELF3* mRNA levels of OC tissues. G,H) Representative immunostaining images of *EHF* (G) and *ELF3* (H) in FUSCC cohort2 OC tissues. I,J) Kaplan–Meier curves of overall survival (I) and disease‐free survival (J) in FUSCC cohort 2 stratified by *EHF* protein levels of OC tissues. K,L) Kaplan–Meier curves of overall survival (K) and disease‐free survival (L) in FUSCC cohort 2 stratified by *ELF3* protein levels of OC tissues. M,N) Kaplan–Meier curves of overall survival (M) and disease‐free survival (N) in FUSCC cohort 2 stratified by *KLF5*/*EHF*/*ELF3* protein levels of OC tissues. ^***^ *p* < 0.001.

Although our results revealed the key role of *KLF5* in promoting OC carcinogenesis, little is known about the clinical significance and molecular functions of *EHF* and *ELF3* in OC patients. RT‐qPCR validated that *EHF* mRNA was significantly overexpressed in OC tissues in FUSCC cohort 1 (**Figure** **7**A; *p* < 0.001). However, there was no significant correlation between EHF expression of *EHF* and overall survival (Figure 7B; *p* = 0.31) or disease‐free survival (Figure 7C; *p* = 0.669) in OC patients. We also found that the mRNA level of *ELF3* was highly expressed in OC samples from FUSCC cohort 1 (Figure 7D; *p* < 0.001), and OC patients with high *ELF3* expression showed a lower overall survival rate (Figure 7E; *p* = 0.012, hazard ratio = 2.151) and disease‐free survival rate (Figure 7F; *p* = 0.026, hazard ratio = 1.773). Accordingly, IHC revealed overexpression of the *EHF* (Figure 7G) and *ELF3* (Figure 7H) proteins in OC tissues compared to normal tissues in FUSCC cohort 2. OC patients with high *EHF* protein expression showed lower overall survival rates (Figure 7I; *p* = 0.015, hazard ratio = 1.618), but there was no significant correlation with disease‐free survival rates (Figure 7J; *p* = 0.053, hazard ratio = 1.397). OC patients with high *ELF3* protein expression showed lower overall survival rates (Figure 7K; *p* = 0.003, hazard ratio = 1.905) and disease‐free survival rates (Figure 7L; *p* = 0.025, hazard ratio = 1.522).

Next, we evaluated the correlation between *KLF5*, *EHF*, and *ELF3* expression at the protein level, and the results showed a significant positive correlation between their protein levels in the OC samples (Figure S7A,B, Supporting Information; *p* < 0.001). The TCGA and GSE2109 datasets also revealed a significant positive correlation between their mRNA levels in OC samples (Figure S7C, Supporting Information). More importantly, OC patients with high *KLF5*, *EHF*, and *ELF3* protein levels showed worse overall survival rates (Figure 7M; *p* < 0.001, hazard ratio = 2.181) and disease‐free survival rates (Figure 7N; *p* < 0.001, hazard ratio = 2.275). These data suggest that *KLF5* combined with *EHF* and *ELF3* may be an effective prognostic factor for patients with OC. These results emphasize that *EHF* and *ELF3* are highly expressed in OC tissues. *KLF5*, combined with *EHF* and *ELF3*, could be a novel and effective prognostic factor for OC patients.

### 2.8 Targeting *KLF5* Increases the Sensitivity of OC Patients to PARPi Resistance

**Figure 8:**
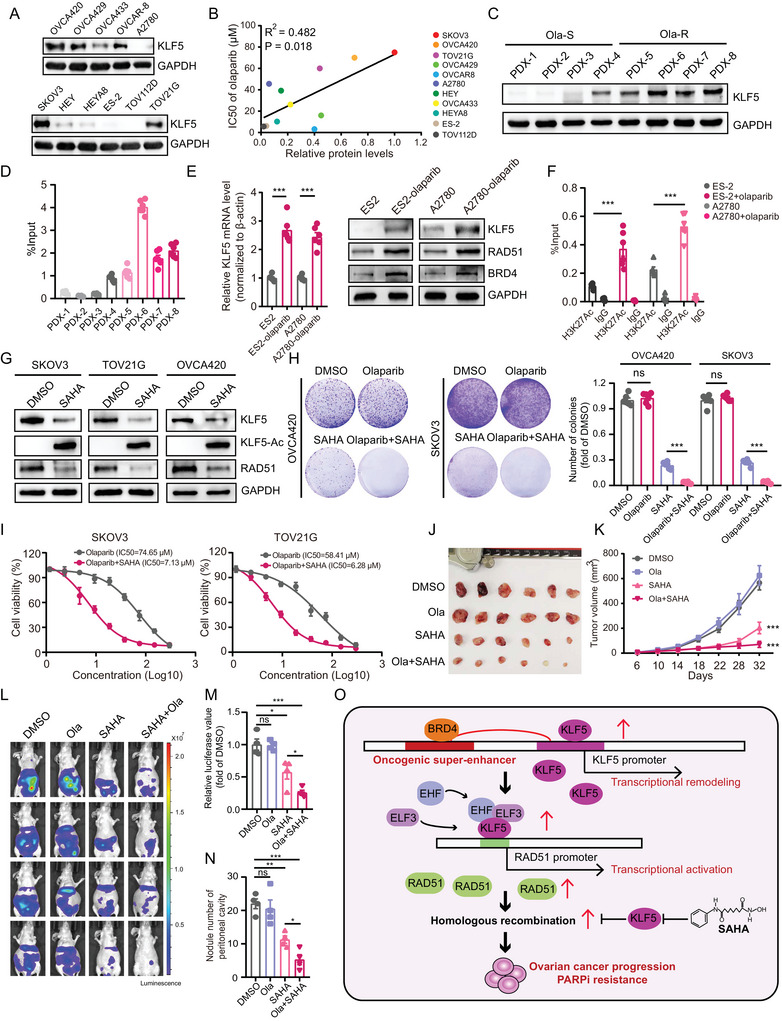
*KLF5* governs OC susceptibility to PARP inhibitors. A) Immunoblotting for *KLF5* protein levels in 11 OC cell lines. B) The correlation between *KLF5* protein levels and IC50 value of olaparib in OC cell lines. C) Immunoblotting for *KLF5* protein levels in olaparib‐sensitive (Ola‐S) or resistant (Ola‐R) PDX samples of OC. D) The activity of super‐enhancer 2 of *KLF5* in olaparib sensitive or resistant PDX samples of OC determined by ChIP‐qPCR of H3K27Ac signal. E) The *KLF5* mRNA levels (left) and *KLF5, RAD51, BRD4* protein levels (right) in ES2, A2780 and olaparib‐treated ES2 and A2780 cells. F) The activity of super‐enhancer 2 of *KLF5* in ES2, A2780, and olaparib‐treated ES2 and A2780 cells was determined by ChIP‐qPCR of the H3K27Ac signal. G) Immunoblotting for *KLF5* and *RAD51* protein levels in SKOV3, TOV21G, and OVCA420 cells treated with DMSO or SAHA (1 µm). H) The colony formation assay in OVCA420 and SKOV3 cells treated with DMSO, olaparib (10 µm), SAHA (0.5 µm), and olaparib combined with SAHA. I) The IC50 value of olaparib in SKOV3 and TOV21G cells treated with olaparib or olaparib combined with SAHA. J,K) Xenograft tumors (J) and tumor volume (K) in NSG mice model treated with DMSO, olaparib, SAHA, and olaparib combined with SAHA. L–N) The luciferase image (L), luciferase value (M), and nodule number of the peritoneal cavity (N) show the abdominal tumor metastasis of TOV21G cells treated with DMSO, olaparib, SAHA, olaparib combined with SAHA in BALB/C‐nude mice. O) The working model of SEs‐driven *KLF5* promoted OC progression and PARPi resistance through “*KLF5*/*EHF*/*ELF3*‐*RAD51*‐HRR pathway”, and SAHA combined with olaparib may be the promising strategy for *KLF5* highly expressed and PARPi‐resistant OC patients. Values represent the mean ± SEM, *n* = 6 in (D–F; H; J–K), *n* = 4 in (L–N). ^*^ *p* < 0.05, ^**^ *p* < 0.01, and ^***^ *p* < 0.001.

Given that HRR is the core pathway of PARPi resistance in tumors, and our previous work demonstrated that *KLF5* regulates HRR by remodeling *RAD51* transcription in OC, we hypothesized that *KLF5* may be involved in PARPi resistance in OC cells. We first verified *KLF5* protein levels in OC cell lines and found that *KLF5* was highly expressed in OVCA420, SKOV3, TOV21G, and other cell lines (**Figure** **8**A). Interestingly, there was a significant positive correlation between *KLF5* protein levels and the IC50 value of olaparib, a PARP inhibitor widely used in clinical treatment (Figure 8B). Similar high *KLF5* protein expression levels were found in four olaparib‐resistant patient‐derived xenografts (PDX) of OC compared with four olaparib‐sensitive PDX samples (Figure 8C). Moreover, SE of No. 2 upstream of *KLF5* showed a higher modified H3K27Ac signal in olaparib‐resistant PDX samples (Figure 8D).

We further constructed olaparib‐resistant cell lines to confirm whether the SEs of *KLF5* drive *KLF5* expression, enhance *RAD51* transcription, and lead to PARPi resistance. Our data showed that *KLF5* and *RAD51* mRNA levels were elevated in olaparib‐resistant ES2 and A2780 cells (Figure 8E; Figure S8A, Supporting Information), and there was a similar protein expression pattern for *KLF5*, *RAD51*, and *BRD4* (Figure 8E). Importantly, olaparib induced the activation of *KLF5* upstream of SE element No. 2 (Figure 8F). The inhibition of the SEs activity of *KLF5* by targeting *BRD4* siRNAs decreased *KLF5* mRNA levels (Figure S8B, Supporting Information), suggesting that PARPi induces the SEs activation of *KLF5* and drives *KLF5* overexpression in OC cells. We also found increased binding of *EHF* and *ELF3* in *RAD51* promoter regions in olaparib‐treated ES2 and A2780 cells (Figure S8C, Supporting Information). To further confirm the biological function of the *KLF5*/*EHF*/*ELF3*‐*RAD51* axis in the regulation of PARPi resistance in OC, we transfected *KLF5* and *RAD51* siRNAs and found that *KLF5* or *RAD51* inhibition decreased colony formation ability in olaparib‐resistant ES2 and A2780 cells (Figure S8D, Supporting Information). Additionally, *KLF5* knockdown combined with olaparib significantly inhibited the colony formation ability of SKOV3 cells (Figure S8E, Supporting Information), whereas overexpression of *KLF5* decreased sensitivity to olaparib (Figure S8F, Supporting Information), and enhanced colony formation (Figure S8G, Supporting Information) in UWB1.289 cells, which carry a BRCA1 mutation and are sensitive to olaparib. Moreover, *RAD51* inhibition reduced the colony formation ability of olaparib‐resistant OC cells (Figure S8H, Supporting Information), and inhibition of *RAD51* (Figure S8I, Supporting Information), *EHF*, or *ELF3* (Figure S8J, Supporting Information) increases the sensitivity of olaparib‐resistant OC cells to olaparib. These results suggest that olaparib induces the SEs activation of *KLF5*, which drives *KLF5* overexpression in OC cells. *KLF5*/*EHF*/*ELF3*‐*RAD51* axis controls PARPi resistance in OC cells.

Studies have reported that the histone deacetylase inhibitor suberoylanilide hydroxamic acid (SAHA) inhibited the protein expression of *KLF5* in breast cancer by increasing *KLF5* protein lysine 369 (K369) acetylation levels.^[^ ^35^ ^]^ Therefore, we explored the regulatory role of SAHA in *KLF5* expression in OC cells. SAHA treatment significantly increased the acetylation levels of *KLF5* and inhibited the total protein levels of *KLF5* and *RAD51* in SKOV3, TOV21G, and OVCA420 cells (Figure 8G). Surprisingly, SAHA combined with olaparib significantly inhibited the proliferation of OVCA420 and SKOV3 cells, which were insensitive to olaparib (Figure 8H). Moreover, SAHA significantly increased the sensitivity of SKOV3 (IC50 = 7.13 µM) and TOV21G (IC50 = 6.28 µM) cells to olaparib (Figure 8I), suggesting a potential role of SAHA in the treatment of OC patients with PARPi resistance and *KLF5* high expression. To verify this function, we constructed an olaparib‐resistant PDX mouse model with *KLF5* high expression and found that olaparib combined with SAHA significantly inhibited subcutaneous tumor formation compared to SAHA or olaparib treatment (Figure 8J,K). The olaparib‐insensitive OC cells TOV21G with *KLF5* high expression were also injected into the abdominal cavity of mice treated with SAHA and olaparib, and we found that olaparib combined with SAHA significantly inhibited the growth and abdominal metastasis of TOV21G cells compared with SAHA or olaparib treatment alone (Figure 8L–N). Kyoto Encyclopedia of Genes and Genomes (KEGG) pathway and GSEA analyses also revealed that SAHA inhibited the HR pathway in OC cells that were insensitive to olaparib (Figure S9, Supporting Information). In summary, these data suggest that patients with PARPi‐resistant OC exhibit abnormal activation of *KLF5* SEs and promote high *KLF5* expression. *KLF5* manipulates olaparib resistance in OC by remodeling *RAD51* transcription and enhancing the HRR pathway. SAHA combined with olaparib may be a potential treatment strategy for PARPi‐resistant OC patients with high *KLF5* expression levels (Figure 8O).

## 3 Discussion

Targeting epigenetic regulatory elements and transcriptomes in low‐mutation tumors such as ovarian and liver cancers has become an effective and feasible approach for developing new treatments. Recent studies have shown that the abnormal activation of epigenetic regulatory elements in OC leads to the remodeling of the tumor transcriptome and promotes growth and metastasis. Abnormal activation of epigenetic regulatory elements such as SEs accelerates OC progression and drug resistance. Yokoyama et al. found that the BET inhibitor JQ1 significantly inhibited the activity of the stemness‐related gene ALDH1A1 in OC, downregulated ALDH1A1 expression, and significantly inhibited the growth of platinum‐resistant OC cells.^[^ ^16^ ^]^ Shang et al. also revealed the critical role of SOX9, driven by SEs, in platinum resistance in OC.^[^ ^11^ ^]^ Kelly et al. analyzed 126 SEs bound to *BRD4* in OVCAR3 OC cells. Importantly, CRISPR‐interference and CRISPR‐deletion systems were used to functionally screen 86 SEs and identify new SEs with oncogenic functions.^[^ ^10^ ^]^ Encouraged by these outstanding results, we further conducted H3K4me1, H3K4me3, H3K27Ac, polII, and EP300 ChIP‐seq experiments in six ovarian cancer cell lines and normal ovarian epithelial cells. The collected H3K27Ac data of six additional OC cell lines from the ENCODE database jointly integrated and depicted the landscape of abnormally activated SEs in OC. Unexpectedly, we discovered two abnormally activated SEs upstream of *KLF5* in OC, and the activity of these two elements was significantly suppressed in normal ovarian epithelial cells. We also confirmed that SEs regulate the transcription of *KLF5*, which has been reported in other tumors.^[^ ^27^ ^]^ Moreover, we found that *KLF5* promotes self‐regulation by binding to its promoter region, further enhancing its transcriptional activity. This partly explains why some OC cells did not exhibit high activity signals of H3K27Ac features for these two SEs, whereas *KLF5* mRNA or protein levels exhibited highly expressed features. The combination of SEs and *KLF5* self‐regulation further emphasizes their specific high expression in OC, suggesting that *KLF5* may be a relatively specific driving factor for OC patients.

Recent studies have demonstrated a regulatory relationship between *KLF5* and SEs in several types of cancer. For example, Jiang et al. showed that *KLF5*, TP63, and SOX2 are core regulatory factors that establish SEs‐regulated circuits, vital for ALDH3A1 transcriptional regulation and ESCC cell viability.^[^ ^26^ ^]^ Chen et al. further validated an interconnected circuitry formed by four master TFs‐*ELF3*, *KLF5*, GATA6, and *EHF*, which promoted each other's expression by interacting with each SE in esophageal cancer.^[^ ^27^ ^]^ Moreover, SE activation drives *KLF5* overexpression and promotes basal‐like breast cancer (BLBC) progression. Inhibition of *KLF5* SEs using *BRD4* and CDK7 inhibitors significantly downregulated *KLF5* expression, suggesting an effective therapeutic strategy for treating BLBC by targeting *KLF5* and its SEs.^[^ ^36^ ^]^ These results highlight not only the role of SEs in *KLF5* expression but also the pivotal role of *KLF5* in establishing SE‐regulated circuits with other core regulatory factors in several cancers. In OC, *KLF5* mainly binds to the promoter regions of targeted genes. *KLF5* forms transcription complexes with *EHF*, and *ELF3* binds to the promoter region of *RAD51*, remodels *RAD51* transcription, and strengthens the HR pathway. During the experiment, we also found that *KLF5* and *BRD4* proteins have a certain binding ability, and knocking down *KLF5* inhibited *BRD4* protein levels but did not affect mRNA levels, suggesting that *KLF5* may affect *BRD4* protein stability and has the potential to regulate SEs in OC. We will further investigate the relationship between *KLF5* and downstream SEs in subsequent studies to expand our understanding of the important role of *KLF5* in the occurrence and progression of OC.

*RAD51* is a key gene for HRR in cells and a core target for PARPi resistance in OC. Fang et al. used ChIP‐qPCR to verify that the inhibition of FOXM1 and CEBPB inhibits the transcription of *RAD51* and enhances the sensitivity of OC to PARP inhibitors.^[^ ^37^, ^38^ ^]^ Currently, there are no reports of the direct observation of TFs in the transcriptional regulation of *RAD51* using ChIP‐seq experiments in OC. In this study, we identified the genome‐wide binding sites of *KLF5* and found, for the first time, that *KLF5* could bind to the *RAD51* promoter and remodel its transcription levels using ChIP‐seq. We also found that *KLF5* binds to the promoter region of other HRR‐related genes, such as *CHEK1*, *RAD54L*, *EME1*, and *BLM*, and the knockdown of *KLF5* significantly inhibited the mRNA levels of these genes. Our work underscores the critical role of *KLF5* in the transcriptional regulation of the HRR pathway. Chen et al. reported interconnected loops formed by *ELF3*, *KLF5*, *GATA6*, and *EHF* in esophageal cancer, which promote their expression by interacting with their SEs.^[^ ^27^ ^]^ The binding of *KLF5* to *EHF* and *ELF3* but not *GATA6* was also observed in the transcriptional protein complex in our study. *EHF* and *ELF3* play important roles as cancer‐promoting TFs in various cancers,^[^ ^39^, ^40^ ^]^ their involvement in HRR has not been reported. We confirmed that *EHF* and *ELF3* were highly expressed in OC samples, whereas GATA6 showed the opposite expression characteristics (GEPIA, http://gepia.cancer‐pku.cn). Knockdown of *EHF* and *ELF3* significantly inhibited *RAD51* transcription. Therefore, the *KLF5*‐*EHF*‐*ELF3* protein complex may be crucial for regulating the HRR pathway in OC. Furthermore, *KLF5* knockdown significantly reduced the binding ability of *EHF* and *ELF3* to the *RAD51* promoter, suggesting that *KLF5* plays a central role in the transcriptional regulation of this protein complex. In our study, we observed that *KLF5* may regulate DNA replication and spliceosome pathways, which are also abnormally altered and critical in cancer. *KLF5* has also been found to be involved in RNA splicing loops to promote tumor progression.^[^ ^41^ ^]^ Whether these pathways mediate the oncogenic function of *KLF5* in OC requires further investigation.

The HRR pathway is important for PARPi resistance in cancers, and targeting the core regulatory targets of HRR is the key to developing effective treatments for PARPi resistance. Both JQ1 and histone deacetylase inhibitors have been reported to increase tumor cell susceptibility to PARP inhibitors.^[^ ^42^, ^43^ ^]^ We further found that SAHA had a stronger inhibitory effect on the expression level of *KLF5* and the growth of OC cell growth than JQ1 at the same drug concentration. Therefore, SAHA was selected for subsequent preclinical experiments. SAHA has been shown to enhance the sensitivity to PARP inhibitors in malignant tumors such as liver cancer and acute myeloid leukemia.^[^ ^44^ ^]^ Treatment with SAHA combined with PARP inhibitors has only been sporadically reported for OC.^[^ ^45^ ^]^ In this study, we used a variety of OC cell lines and PDX models with high *KLF5* expression to confirm the treatment efficiency and mechanism of SAHA in inhibiting the transcription of *RAD51*. Importantly, we found that SAHA downregulated *RAD51* transcription mainly by inhibiting *KLF5* expression, and SAHA combined with olaparib was identified as a potential treatment for PARPi resistance in OC patients with high *KLF5* expression. It is worth noting that we have identified that *KLF5* is regulated by super‐enhancers and self‐regulation in OC, resulting in the formation of two subgroups: the super‐enhancer‐driven *KLF5* ovarian cancer subgroup and the self‐regulatory *KLF5* subgroup. This epigenetic transcriptional regulation heterogeneity necessitates a stratified approach and the use of different therapeutic strategies for targeting *KLF5* in the clinical setting. Based on our previous findings that the super‐enhancer‐targeting drug JQ1 can inhibit KLF5 transcription and its potential therapeutic effect in ovarian cancer, as well as its role in enhancing PARPi sensitivity, we propose that a combination treatment of JQ1 and olaparib can be considered for OC patients with super‐enhancer‐driven *KLF5* and concurrent PARPi resistance. For OC patients with self‐regulatory *KLF5* and concurrent PARPi resistance, a combination treatment of SAHA and olaparib may be suitable. We have also demonstrated that SAHA targets the acetylation modification of *KLF5* protein in OC, leading to reduced total protein levels of *KLF5* and exerting anticancer effects. Therefore, the combination of SAHA and olaparib also represents a potentially effective treatment option for OC patients with super‐enhancer‐driven KLF5 and concurrent PARPi resistance.

We also performed ChIP‐seq assays for H3K4me1, H3K4me3, and polII in OC cells and identified genome‐wide modifications or binding sites. Therefore, these data can be further mined to identify other abnormally activated regulatory elements, such as promoters, enhancers, and corresponding transcriptional regulatory networks. As our previous data showed that OC produces the most TSTs, a considerable portion of TSTs are transcribed from abnormally activated promoter elements.^[^ ^46^ ^]^ For example, tumor transposon elements can act as highly active promoters after apparent activation. It can directly transcribe tumor‐addictive or tumor‐specific transcripts and hijack the high expression of adjacent oncogenes to promote cancer progression and drug resistance.^[^ ^5^, ^9^, ^47^ ^]^ At present, the biological functions and transcriptional regulatory mechanisms of most TSTs in OC remain unclear. In the future, we will use these apparent data to integrate the OC transcriptome to explore further the relationship between epigenetic regulatory elements, such as promoters and TSTs, and to provide new targets and molecular mechanisms for developing drug resistance in OC patients.

In summary, we depicted the landscape of abnormally activated SEs in OC and identified two super‐enhancers that regulate *KLF5* transcription, resulting in *KLF5* self‐transcriptional regulation. *KLF5* forms a transcription complex with *EHF* and *ELF3*, remodels *RAD51* transcription, strengthens the HRR pathway in OC cells, and ultimately promotes OC progression and PARPi resistance. Based on our preclinical results, we suggest that the HDACi inhibitor SAHA can repress *KLF5* expression and that SAHA combined with olaparib may be a new potential treatment for PARPi‐resistant OC patients with high *KLF5* expression levels. As the landscape of SEs in OC and their role in PARPi resistance have not been previously described, our study provides an opportunity for epigenetic regulatory elements to remodel the tumor transcriptome for their contribution to tumor progression, diagnosis, and treatment.

## 4 Experimental Section

### Study Approval

All animal experiments were performed in accordance with the guidelines of the Animal Care and Use Committee of the FUSCC. The animals were treated in compliance with relevant institutional and national regulations and guidelines. The maximum allowed tumor size/burden did not exceed a diameter of 1.5 cm. Two OCs cohorts were included in the study. In FUSCC cohort 1, 72 normal ovarian tissue and 80 OC tissue samples were collected, and RNA was extracted and reverse‐transcribed into cDNA. In FUSCC cohort 2, 74 normal ovarian tissue and 165 OC tissue samples were collected and a tissue microarray was constructed. Normal ovarian tissue was obtained during surgery for other gynecological diseases in FUSCC. All OC tissue samples were obtained from patients in FUSCC, and written informed consent was obtained. The Institutional Review Board of Fudan University approved the use of human specimens. The assigned approval numbers of the investigator for using human specimens is 050432‐4‐2108*, and the number of performing animal experiments is FUSCC‐IACUC‐2021400 and FUSCC‐IACUC‐2023215.

### Cell Culture

Human OC cell lines TOV21G, OVCAR3, and SKOV3 cells were obtained from the American Type Culture Collection (ATCC, Manassas, Virginia, USA). A2780, OVCA420, OVCA429, ES‐2, IOSE80, and human embryonic kidney 293T cell line (HEK293T) used in this study were purchased from Shanghai Cell Bank Type Culture Collection (Shanghai, China). All cell lines were cultured in DMEM (#L110, BasalMedia, Shanghai, China) medium supplemented with 10% FBS (#10099‐141, Gibco, CA, USA) and 1% penicillin‐streptomycin (#S110B, BasalMedia), and were grown in a humidified environment consisting of 95% air and 5% CO_2_ at 37 °C. All these cell lines were mycoplasma‐free tested using an RT‐qPCR‐based method and authenticated by short tandem repeat (STR) profiling and cell vitality.

### Antibodies

The antibodies against H3K4me1 (39 635; ChIP‐seq), H3K27ac (39 133; ChIP‐qPCR and ChIP‐seq), RNApol II (61 667; ChIP‐qPCR and ChIP‐seq) and p300 (61 401; ChIP‐seq) were purchased from Active Motif (Carlsbad, CA, USA); H3K4me3 (9751; ChIP‐seq), *KLF5* (51586s; ChIP‐seq, WB, IHC, IP) and Phospho‐Histone H2A.X (97 148; WB) were from Cell Signaling Technology (Danvers, MA, USA); Flag‐tag (F1804; ChIP‐qPCR) were from Sigma‐Aldrich; *BRD4* (A301‐985A50; ChIP‐seq, WB) were from Thermo Fisher Scientific. *EHF* (PA5‐63890; WB, IHC, co‐IP), KLF5‐K369 acetylation were generated by GenScript (Nanjing, China). ELF3 (0 03479; WB, IHC, co‐IP) was from Sigma–Aldrich (St. Louis, MO, USA) and *ELF3* (A5236, WB, co‐IP) was from ABclonal (Wuhan, China). *RAD51* (14961‐1‐AP; WB) and GAPDH (60004‐1‐Ig; WB) were from Proteintech (Wuhan, China).

### Vector Construction

The open reading frames (ORFs) of *EHF* and *ELF3* were amplified from HEK293T cell cDNA and cloned into pCDH‐CMV‐3X‐Flag‐Puro (SBI, Palo Alto, CA, USA) using Seamless Cloning and Assembly Kit (Transgen Biotech, Beijing, China). For enCRISPRi assay, pHR‐SFFV‐KRAB‐dCas9‐P2A‐mCherry and Lenti_MCP‐LSD1_Hygro plasmids were purchased from Addgene (Watertown, USA). sgRNA sequences targeting SEs of *KLF5* were designed by CRISPR‐ERA (http://crispr‐era.stanford.edu/) and inserted into the Lenti_sgRNA(MS2)_ZsGreen1 (Addgene) using BsmbI (NEB). sgRNA sequences targeting *KLF5* protein‐coding regions were designed by CRISPR‐ERA (http://crispr‐era.stanford.edu/) and inserted into Lenti‐guide‐puro (Addgene) using BsmbI. The *RAD51* promoter sequence containing the *KLF5* binding site was amplified from HEK293T cell cDNA and cloned into PGL3‐enhancer (Progema, Madison, USA). The sgRNA sequences are provided in Table S7 (Supporting Information).

### RNA Interference

The siRNA oligonucleotides targeting *KLF5*, *ELF3*, *EHF*, *BRD4*, and the negative control were designed and synthesized by RiboBio (Guangzhou, China). Cells were transfected with the indicated siRNAs or control oligos using the Lipofectamine RNAiMAX reagent (Invitrogen, Carlsbad, CA, USA) at a final concentration of 50 nm. After transfection for 48 h, the cells were used for further experiments, such as transwell migration, RNA extraction, and immunoblotting assays. The sequences for the gene‐specific siRNAs used are listed in Table S7 (Supporting Information).

### Plasmid Transfection and Lentiviral Infection

Transient plasmid transfection was performed using Lipofectamine 2000 according to the manufacturer's protocol. To generate stable cell lines, HEK293T cells were transfected with corresponding expression plasmids together with packaging plasmid (pMD2G and psPAX2). And the supernatant containing viruses was collected and filtered through 0.45 µm filters (FPV403030, JET BIOFIL, Guangzhou, China) after 48 h of transfection, and used for infecting target cells in the presence of 8 µg mL^−1^ polybrene (H9268, Sigma–Aldrich, St. Louis, MO, USA) or stored at −80 °C. After another 48 h, infected cells were further selected by 2 mg mL^−1^ of puromycin (13884‐500, Cayman Chemical, Ann Arbor, MI, USA).

### RNA Extraction and Real‐Time Quantitative PCR (RT‐qPCR)

Total RNA from the tissue specimens or cell lines were extracted with TRIzol reagent (Invitrogen) according to the manufacturer's protocol, resuspended in RNase‐free water, and quantified by NanoDrop spectrophotometer (Thermo Fisher Scientific, Carlsbad, California, USA). cDNA was transcribed by the PrimeScript RT Reagent Kit (TaKaRa, Shiga, Japan) and the quantitative real‐time polymerase chain reaction (qRT‐PCR) was performed with SYBR Green Premix Ex Taq (Takara). Relative RNA expression levels determined by qPCR were measured by ABI Prism 7900 sequence detection system (Applied Biosystems, MA, USA). β‐Actin was used as the internal control and all primers used for qPCR assays are listed in Table S7 (Supporting Information).

### Co‐Immunoprecipitation (co‐IP) and Mass Spectrometry

A sample of ≈1 × 10^7^ OVCA420 cells was lysed with co‐IP lysis buffer (20 mm Tris‐HCl pH 7.5, 150 mm NaCl, 0.5% NP‐40, 10% glycerol, 1 mm MgCl2, 1 mm EDTA, 1X proteinase inhibitor cocktail) and centrifuged at 13 000 rpm for 8 min. The supernatants were collected and incubated with 5 µg *KLF5* specific antibodies and control IgG followed by the addition of 50 µL of Protein G Dynabeads (Thermo Fisher Scientific). After overnight incubation at 4 °C, the beads were washed three times with co‐IP lysis buffer and boiled for 5 min in 50 µL of SDS loading buffer for Western blotting analysis. The eluted proteins were subjected to silver staining by SDS‐PAGE. The electrophoresis band was cut and recovered when it ran to 1 cm and was excised for proteomics screening by mass spectrometry analysis (Shanghai Applied Protein Technology, Shanghai, China). Protein identification was retrieved by Mascot version 2.4.01 (Matrix Science, London, UK), in the human RefSeq protein database (National Center for Biotechnology Information).

### Chromatin Immunoprecipitation Sequencing (ChIP‐seq) and ChIP‐qPCR

OV cells were cross‐linked in 1% formaldehyde, quenched in 125 mm glycine for 5 min, re‐suspended in ChIP lysis buffer (25 mm Tris‐HCl pH 7.5, 500 mm NaCl, 1% Triton X‐100, 0.1% sodium deoxycholate, 0.05% SDS, 1 mm EDTA, 1X proteinase inhibitor cocktail) and sonicated with a Bioruptor UCD‐200 (Diagenode, Liege, Belgium). Solubilized chromatin was immunoprecipitated with antibodies against H3K4me1, H3K4me3, H3K27Ac, polII, EP300, *BRD4*, *KLF5* on Dynabeads Protein G (Thermo Fisher Scientific). The bound fractions were washed four times with ChIP lysis buffer. DNA fragments were digested by RNase A, and proteinase K and recovered by MinElute Reaction Cleanup Kit (Qiagen, Hilden, Germany). Sequencing was performed on an Illumina HiSeq (San Diego, CA, USA). Reads were aligned to hg38 using bowtie2. ChIP‐qPCR was performed on an ABI 7900HT Fast Real‐Time PCR System (Thermo Fisher Scientific) using SYBR Premix Ex Taq II (TaKaRa). ChIP‐qPCR primers used were specified in Table S7 (Supporting Information).

### Super‐Enhancers Identification

ROSE software (https://hpc.nih.gov/apps/ROSE.html) was employed to analyze the H3K27Ac ChIP‐seq data from 12 ovarian cancer cell lines and to identify super‐enhancers. The ChIP‐seq peaks of H3K27Ac were detected using a peak‐finding algorithm MACS. The genomic regions that were enriched with H3K27ac signals and located far from the transcription start site (TSS) of genes (2.5 kb upstream/downstream of TSS) were defined as active enhancers. These enhancers were stitched together if peaks within 12.5 kb of one another, and ranked by their difference in H3K27ac signal versus input signal. Enhancers were plotted with enhancer rank versus enhancer density, and all enhancer regions above the inflection point of the curve were defined as SEs. Super‐enhancers and typical enhancers were assigned to the genes using the default parameters of the ROSE algorithm.

### Luciferase Assays

OVCA420 and OVCAR3 cells were seeded in 96‐well plates at a density of 5000 cells per well and transfected with *KLF5*, *BRD4*, *EHF*, *ELF3* siRNA mix, and siRNA control after cell attachment. Then, a mixture of 50 ng PGL3‐*RAD51*‐enhancer and 10 ng *Renilla* plasmids were transfected. After 48 h, Firefly and *Renilla* luciferase activities were measured by the Dual‐Luciferase Reporter Assay System (Promega, Madison, Wisconsin, USA). The relative firefly luciferase activities were detected while *Renilla* luciferase activities were used as an internal control.

### RNA Sequencing

The total RNA of OC tissues or OVCA420 cells was extracted using TRIzol. Before RNA library construction, rRNAs were removed using the RiboMinus Eukaryote kit (Qiagen, Valencia, CA, USA). RNA samples were fragmented, and cDNA was synthesized using random hexamer primers. cDNA ends were repaired using the End‐It DNA End Repair kit, and A was added to the 3′ end. cDNA fragments were then ligated to adaptor sequences, treated with uracil DNA glycosylase, purified and subjected to quality control using a Bioanalyzer 2100 (Agilent, Santa Clara, CA, USA) and sequenced using a HiSeq 3000 (Illumina, San Diego, CA, USA). Sequencing reads were aligned to the human reference genome (hg38) using the splice‐aware aligner HISAT2 and normalized into FPKM (fragments per kilo base of transcript per million mapped reads) values (FPKM ≥ 0.1).

### Cell Proliferation, Colony Formation, and Migration Assays

Cell proliferation was measured using the Cell Counting Kit‐8 (CCK8) assay (Dojindo Laboratories, Kumamoto, Japan) as described in the manufacturer's manual. For colony formation assays, 2000 OC cells were seeded in 6‐well plates in triplicate and cultured under normal growth conditions for nearly 8–12 days. The colonies were fixed and stained with 100% methanol and a dye solution containing 0.5% crystal violet (Sigma–Aldrich, Missouri, USA). The number of colonies was counted and analyzed. For the Transwell migration assay, Boyden chambers with 8 µm pores (Corning, New York, NY, USA) were applied. Two hundred macroliters serum‐free growth medium containing 3 × 10^4^ cells was added in the upper chamber while medium containing 10% FBS, acting as a chemoattractant, was added in the lower chamber simultaneously. After 24 h of incubation, migrated cells were stained with 100% methanol and dye solution containing 0.5% crystal violet, followed by imaging and counting under an inverted microscope (Olympus, Tokyo, Japan).

### Comet Assay

Comet assay was performed using a comet assasy kit (Abcam, Cambridge, UK). Slides were pre‐coated by 1% normal melting agarose. About 1  ×  10^5^ cells were resuspend in ice‐cold PBS, mixed with agarose at a 1/10 ratio, transferred 75 µL per well onto the top of the comment agarose base layer, lysed in pre‐chilled lysis buffer for 1 h at 4 °C in the dark, performed alkaline electrophoresis, cleared with PBS and stained with diluted Vista Green DNA Dye. Finally, the individual cell was observed through epifluorescence microscopy (Olympus, Japan).

### Tissue Microarray (TMA) and Immunohistochemistry (IHC) Evaluation

For TMA, specimens were collected and embedded in paraffin blocks, which were cut into 4 µm sections and subjected to IHC staining. Protein expression of *KLF5* (clone 51586s, CST, 1:300 dilution), *EHF* (clone PA5‐63890, Sigma, 1:300 dilution), and *ELF3* (clone 0 03479, Sigma, 1:300 dilution) on stained slides were assessed by two independent pathologists. For each marker (*KLF5*, *EHF*, and *ELF3*), the cutoff for positivity was decided according to the staining pattern and intensities on all images. All quantifications were evaluated blinded to patient clinical outcomes. The staining percentage and intensity were graded as follows: 0 (0–4%), 1 (5–24%), 2 (25–49%), 3 (50–74%), or 4 (≥75%); and 0, 1, 2, or 3, respectively. The final scores were calculated by multiplying the percentage and intensity scores, which were considered as negative (−), weakly positive (+), moderately positive (++), and strongly positive (+++), corresponding to 0, 1–4, 5–8, and 9–12 final scores, respectively.

### IC50 Assays

For drug sensitivity assays, human OC cell lines were seeded in 96‐well flat‐bottom plates (3000 cells in 100 µL of cell suspension per well) and then exposed to olaparib or combined with SAHA for 3 days at the indicated concentrations to determine the inhibitor concentration that resulted in 50% inhibition of cell viability. Cell viability was estimated using Cell Counting Kit‐8 (Yeasen, Shanghai, China), and the surviving fraction was calculated. SAHA (HY‐10221) and olaparib (HY‐10162) were purchased from MedChemExpress (Shanghai, China).

### In Vivo Assays

Female athymic BALB/c‐nude mice, aged 4–6 weeks were used. For tumorigenesis assay, 5 × 10^6^ *KLF5* knock‐down and control SKOV3 cells were resuspended in 0.2 mL sterile PBS and injected subcutaneously into the flanks of randomly selected mice (*n* = 5 per group). Tumor volumes were measured every 5 days after the appearance of tumors and calculated by the formula (length×width2)/2. After 4 weeks, the mice were sacrificed, and the tumors were harvested, weighed, and recorded.

For the intraperitoneal injection model, 2 × 10^6^ cells of control or *KLF5* knock‐down TOV21G cells in 100 µL PBS were injected into the abdominal cavity of two groups of BALB/c‐nude mice, respectively. After 4 weeks, mice were intraperitoneally injected with 10 µL of D‐luciferin (15 µg µL^−1^) g^−1^ of body weight for the in vivo imaging analysis, anesthetized, imaged using an In Vivo Imaging System (IVIS) Lumina system (Xenogen, MA, USA). Mice were further sacrificed and the numbers of metastatic nodules were counted.

For drug exploration in this study, PARPi‐resistant PDX of OC with high *KLF5* expression was transplanted subcutaneously of NSG mice. After 3 weeks, the mice were randomly assigned into 4 groups (*n* = 6 per group) and treated with DMSO (30 mg kg^−1^), olaparib (30 mg kg^−1^), SAHA (30 mg kg^−1^) or combined usage of olaparib and SAHA. Stable luciferase‐labeled TOV21G cells were generated first and 2 weeks after intraperitoneal injection of BALB/c‐nude mice, the mice were randomly assigned into 4 groups (*n* = 4 per group) based on the different treatment regimens: DMSO (30 mg kg^−1^), olaparib (30 mg kg^−1^), SAHA (30 mg kg^−1^) and combined usage of Olaparib and SAHA. Tumor progression was monitored by assessment of the tumor volume and tumor weight. And the development of peritoneal metastases was monitored as described above.

### Statistical Analysis

All data were presented as the mean ± standard error of the mean from at least three independent experiments. Statistical analyses were performed using IBM SPSS 23.0 and GraphPad Prism 8.0 software. The significance of differences between groups was estimated using the Student's *t*‐test, chi‐square (*χ*2) test, or Wilcoxon test, as appropriate. The probability of survival was estimated using the Kaplan–Meier method, and differences between groups were evaluated using the log‐rank test. Overall survival was defined as the time from surgery to death or the last follow‐up, and disease‐free survival was defined as the time from surgery to recurrence or any reason for death. Risk factors for prognosis were determined using univariate and multivariate Cox regression analyses. All statistical analyses were performed using two‐tailed *p* values, and the statistical significance threshold was set at 0.05 if not explicitly mentioned (^*^ *p* < 0.05, ^**^ *p* < 0.01, and ^***^ *p* < 0.001).

## Conflict of Interest

The authors declare no conflict of interest.

## Author Contributions

Y.W., S.Y.C., and Y.S. contributed equally to this work. Z.X.H., S.L.H., and X.H.W. designed and supervised this study. Z.X.H., Y.W., S.Y.C., Y.S., Y.S., Y.H., and J.C.W. conducted experiments. Z.X.H., Y.W., Q.L., S.Y.C., X.J.C., X.Z.J., J.Z., Z.Z., and H.W. performed data analysis. Y.W. and S.Y.C. collected the OC samples and clinical information. Y.W. and Z.X.H. wrote the manuscript with input from all the authors.

## Supporting information

Supporting Information

Supporting Information

## Data Availability

Research data are not shared.
