# Supplementary material for: KLF5 Promotes Tumor Progression and Parp Inhibitor Resistance in Ovarian Cancer
Source: Adv Sci (Weinh). 2023 Sep 13;10(31):2304638. doi: 10.1002/advs.202304638 (PMC10625120; doi:10.1002/advs.202304638)
Supplement: Supplementary file 1 — Supporting Information [file ADVS-10-2304638-s001.pdf]

ADVANCED SCIENCE
Open Access

Supporting Information

for *Adv. Sci.*, DOI 10.1002/advs.202304638

KLF5 Promotes Tumor Progression and Parp Inhibitor Resistance in Ovarian Cancer

Yong Wu, Siyu Chen, Yang Shao, Ying Su, Qin Li, Jiangchun Wu, Jun Zhu, Hao Wen, Yan Huang, Zhong Zheng, Xiaojun Chen, Xingzhu Ju, Shenglin Huang, Xiaohua Wu* and Zhixiang Hu**

Supplementary Materials

KLF5 promotes tumor progression and PARP inhibitor resistance in ovarian cancer

Yong Wu[1, 2, *], Siyu Chen[1, 2, *], Yang Shao[1, 2, *], Ying Su[1, 2], Qin Li[1, 2], Jiangchun Wu[1], Jun Zhu[1], Hao Wen[1], Yan Huang[1], Zhong Zheng[1], Xiaojun Chen[1], Xingzhu Ju[1], Shenglin Huang[1, 2, †], Xiaohua Wu[1, 2, †], Zhixiang Hu[1, 2, †]

[1]Department of Gynecologic Oncology, Fudan University Shanghai Cancer Center, and Shanghai Key Laboratory of Medical Epigenetics, International Co-laboratory of Medical Epigenetics and Metabolism, Institutes of Biomedical Sciences, Shanghai Medical College, Fudan University, Shanghai, China.

[2]Department of Oncology, Shanghai Medical College, Fudan University, Shanghai, China.

* These authors contributed equally to this work.

† **Correspondence**: Zhixiang Hu, Ph.D., Email: zhixiang_hu@fudan.edu.cn, Xiaohua Wu, M.D., Email: wu.xh@fudan.edu.cn, and Shenglin Huang, Ph.D., Email: slhuang@fudan.edu.cn. Department of Gynecologic Oncology, Fudan University Shanghai Cancer Center, and Shanghai Key Laboratory of Medical Epigenetics, International Co-laboratory of Medical Epigenetics and Metabolism, Institutes of Biomedical Sciences, Shanghai Medical College, Fudan University, 305 Rm., 7# Bldg., 270 Dong An Rd., Shanghai, 200032, China;

Supplementary Figures

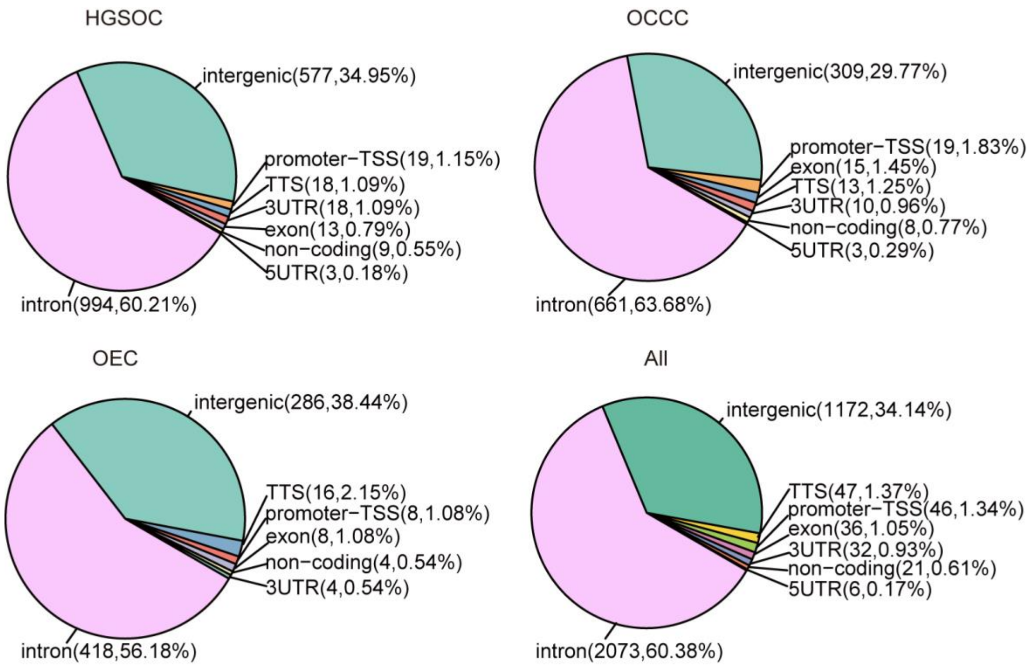

Fig. S1. The number and genomic distribution of super-enhancers in ovarian cancer. 1,651, 1,038 and 744 SEs were identified in HGSOC, OCCC and OEC types of OC respectively, and most of SEs were located in intron and intergenic genomic regeions.

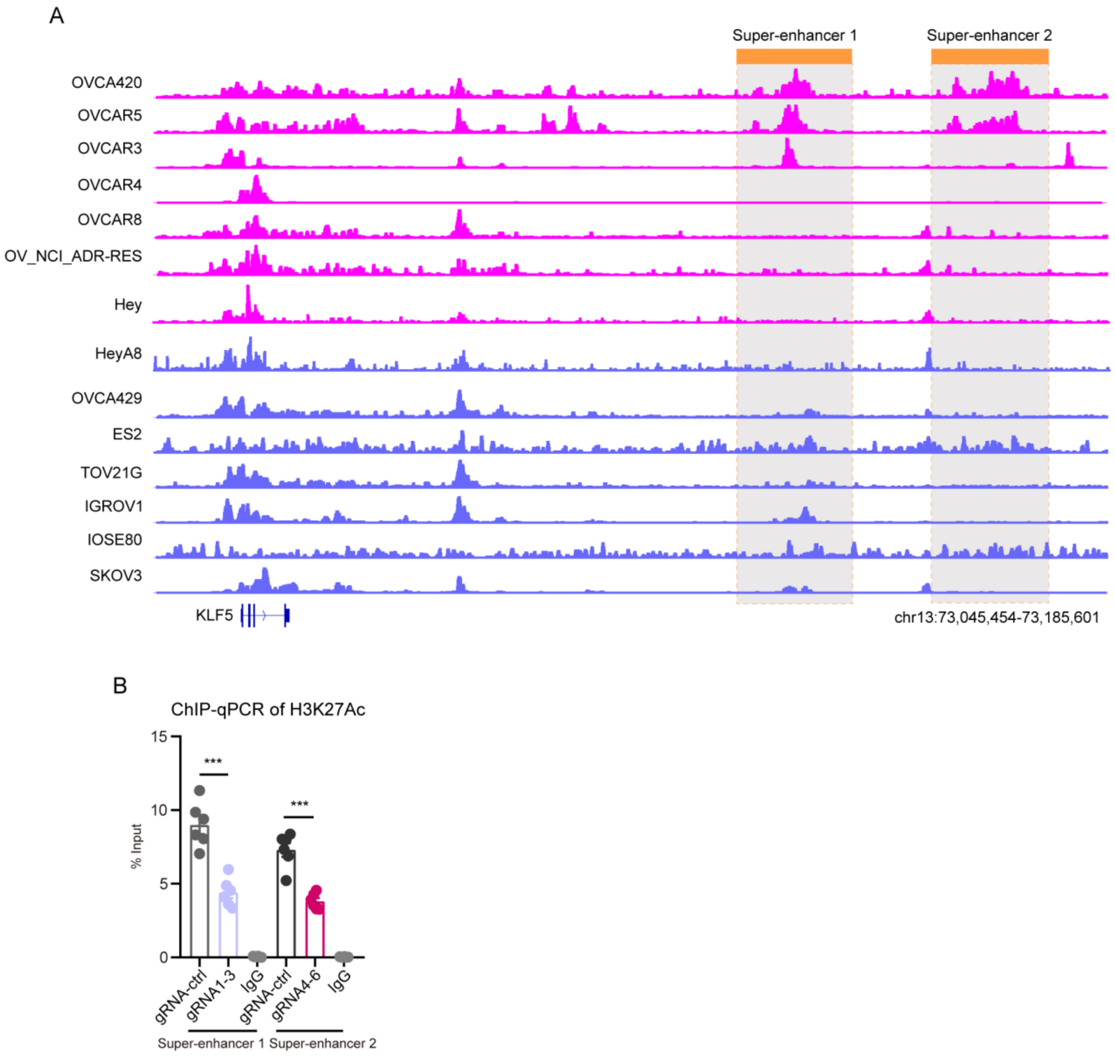

Fig. S2. H3K27Ac signal in *KLF5* SEs and promoter regions. (A) H3K27Ac ChIP-seq of 13 ovarian cancer cell lines and 1 normal ovarian epithelial cells IOSE80 were collected and analysed. SEs were activated in ovarian cancer cells such as OVCA420 and OVCAR5 and were suppressed in IOSE80 cells. (B) pHR-SFFV-KRAB-dCas9-P2A-mCherry, lenti_MCP-LSD1 and lenti_sgRNA(MS2)_ZsGreen1 plasmids targeting SEs of *KLF5* were packaged into lentiviruses. OVCA420 cells were infected with lentiviruses and collected to perform ChIP-qPCR of H3K27Ac. Values represent the mean ± SEM, n=6. ***P < 0.001.

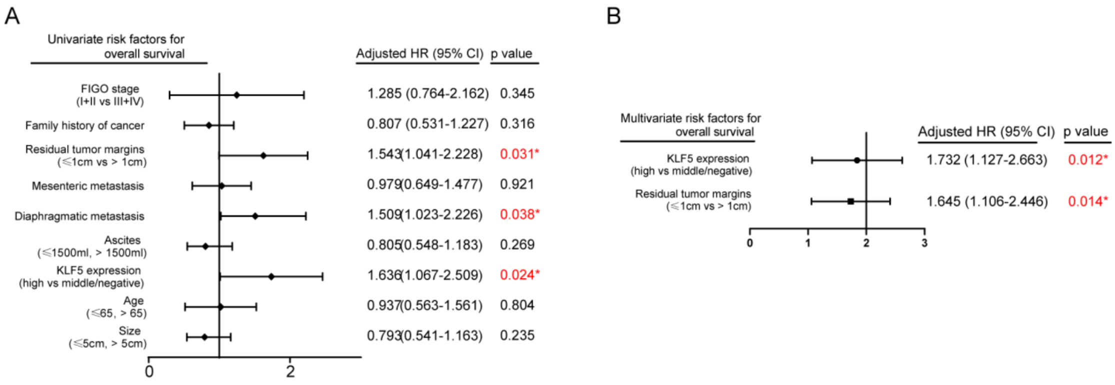

Fig. S3. The correlation between *KLF5* expression levels and clinical significance. (A) The correlation between *KLF5* expression and clinical prognosis of ovarian cancer was analyzed by univariate analysis. (B) The correlation between *KLF5* expression and clinical prognosis of ovarian cancer was analyzed by multivariate analysis.

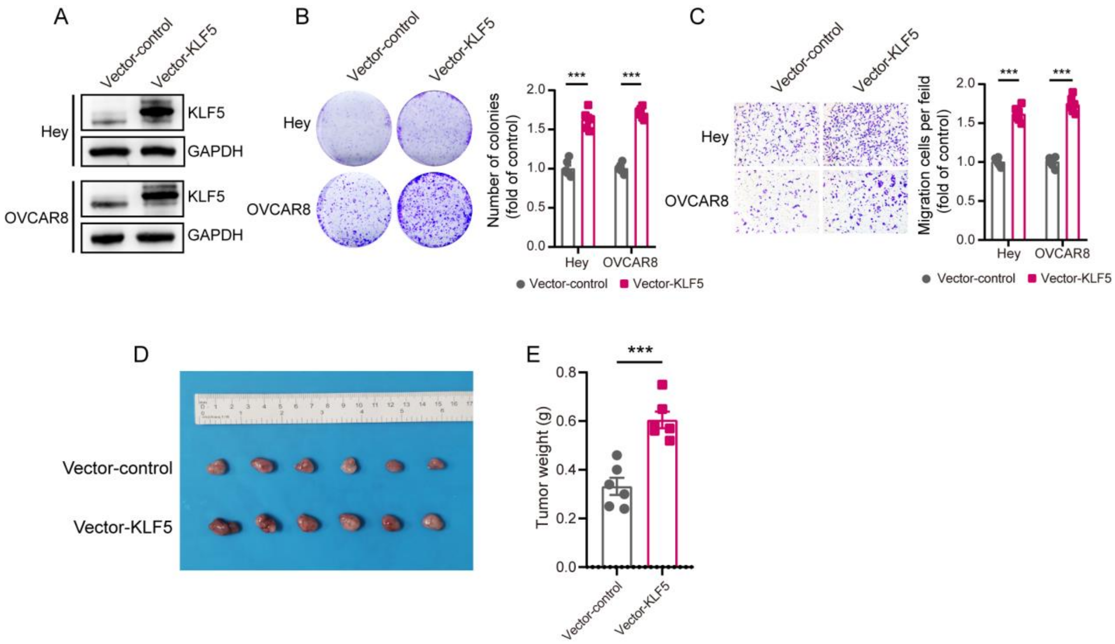

Fig. S4. Overexpression of *KLF5* enhances OC progression. (A) Immunoblotting for *KLF5* protein levels in Hey and OVCAR8 cells infected with PCDH-3XFlag-control or PCDH-3XFlag-*KLF5* lenti-viruses. (B-C) Colony formation assays (B) and transwell migration assays (C) in Hey and OVCAR8 OC cells infected with PCDH-3XFlag-control or PCDH-3XFlag-*KLF5* lenti-viruses. (D) Xenograft tumors of PCDH-3XFlag-control group and PCDH-3XFlag-*KLF5* lenti-viruses group in nude mice. (E) Overexpression of *KLF5* enhanced the weight of xenograft tumors (n=6 mice per group). Values represent the mean ± SEM, n=6 in (B-E). ***P < 0.001.

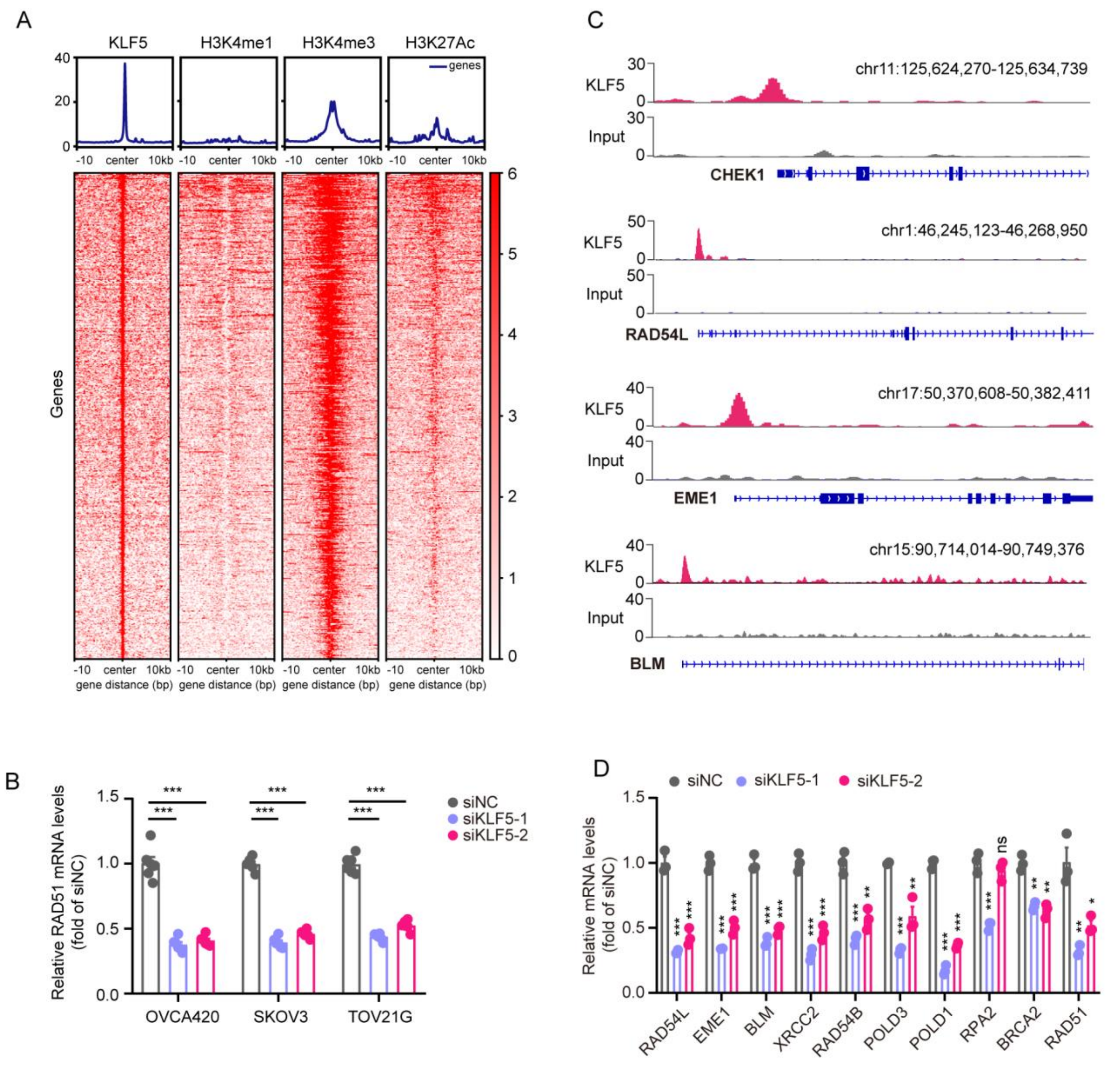

Fig. S5. *KLF5* remodel HR pathway in OC. (A) The enrichment of *KLF5* in TSS, H3K4me1, H3K4me3 and H3K27Ac gemonic regions. (B) The mRNA levels of *RAD51* in OVCA420, SKOV3 and TOV21G OC cells transfected with siNC or si*KLF5* siRNAs. (C) The enriched signal of *KLF5* on the promoter region of the core gene of the homologous recombination repair pathway using *KLF5* ChIP-seq data. (D) The mRNA levels of HR-related genes in OVCA420 cells transfected with siNC or si*KLF5* siRNAs. Values represent the mean ± SEM, n=6 in (B) and n=3 in (D). * P<0.05; **P<0.01; ***P < 0.001; ns: no significant.

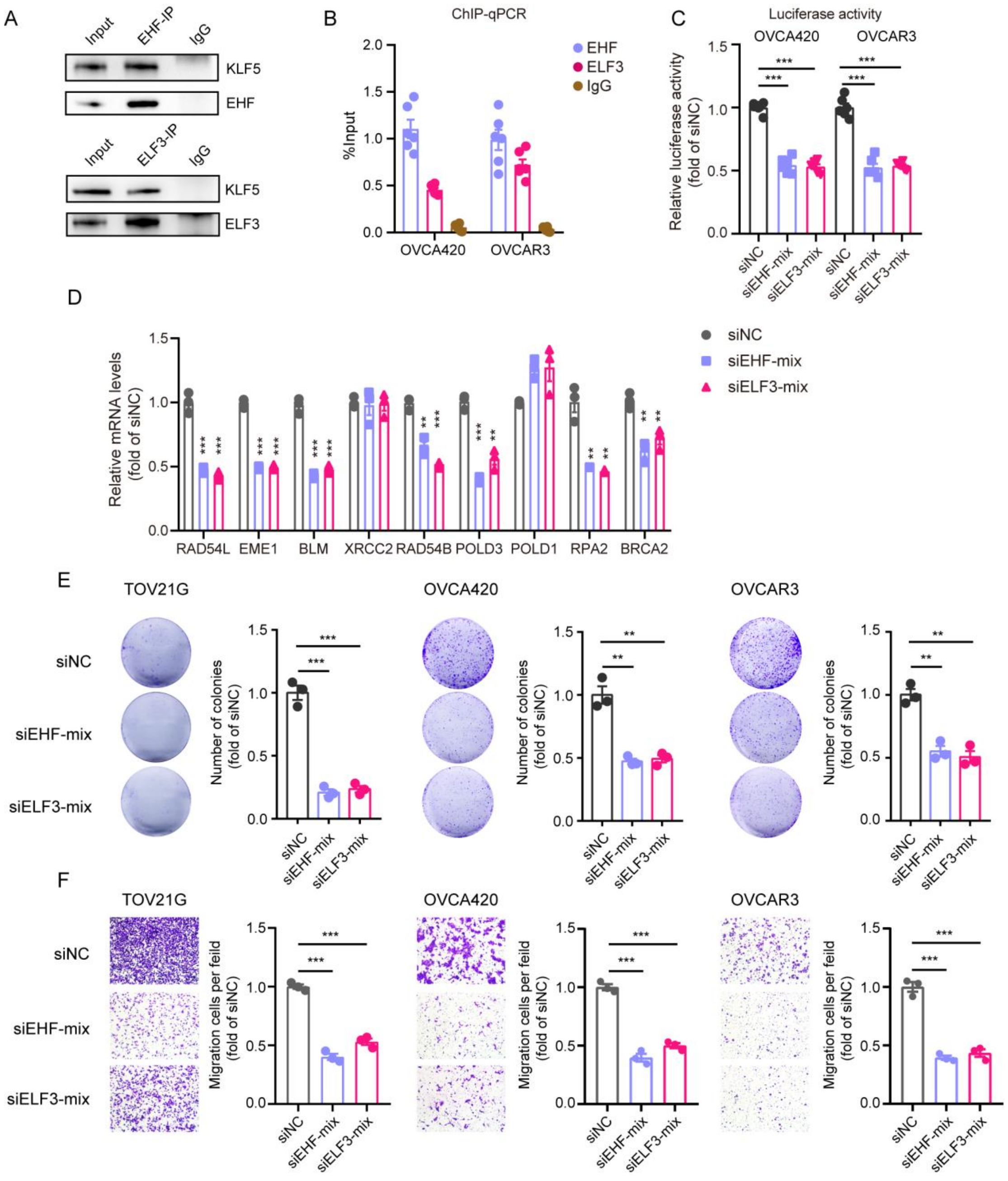

Fig. S6. *EHF* and *ELF3* regulate HR pathway and drive ovarian cancer growth and metastasis in vitro. (A) The co-IP assay of *EHF* and *ELF3* suggesting the binding with *KLF5* in OVCA420 cells. (B) The ChIP-qPCR assay of *EHF* and *ELF3* in *RAD51* promoter regions in OVCA420 and OVCAR3 OC cells. (C) The luciferase activity of *RAD51* promoter regions in OVCA420 and OVCAR3 OC cells transfected with siNC or si*EHF* and si*ELF3* mixed siRNAs. (D) The mRNA levels of HR-related genes in OVCA420 cells transfected with siNC or si*EHF* and si*ELF3* mixed siRNAs. (E) Colony number formation in TOV21G, OVCA420 and OVCAR3 cells transfected

with control, *EHF* and *ELF3* siRNAs. (F) Transwell assay in TOV21G, OVCA420 and OVCAR3 cells transfected with control, *EHF* and *ELF3* siRNAs. Values represent the mean ± SEM, n=6 in (B-C), n=3 in (D-F). **P < 0.01, ***P < 0.001.

A

KLF5 expression	ELF3 expression: n	m	h	total
n	5 (3.0%)	8 (4.8%)	0 (0%)	13 (7.9 %)
m	0 (0%)	38 (23%)	18 (10.9%)	56 (33.9 %)
h	0 (0%)	6 (3.6%)	90 (54.5%)	96 (58.2 %)
total	5 (3.0%)	52 (31.5%)	108 (65.5%)	165
R=0.755				p<0.0001

B

KLF5 expression	EHF expression: n	m	h	total
n	1 (0.6%)	8 (4.8%)	4 (2.4%)	13 (7.9%)
m	0 (0%)	37 (22.4%)	19 (11.5%)	56 (33.9%)
h	3 (1.8%)	23 (13.9%)	70 (42.4%)	96 (58.2%)
total	4 (2.4%)	68 (41.2%)	93 (56.4%)	165
R=0.335				p<0.0001

C

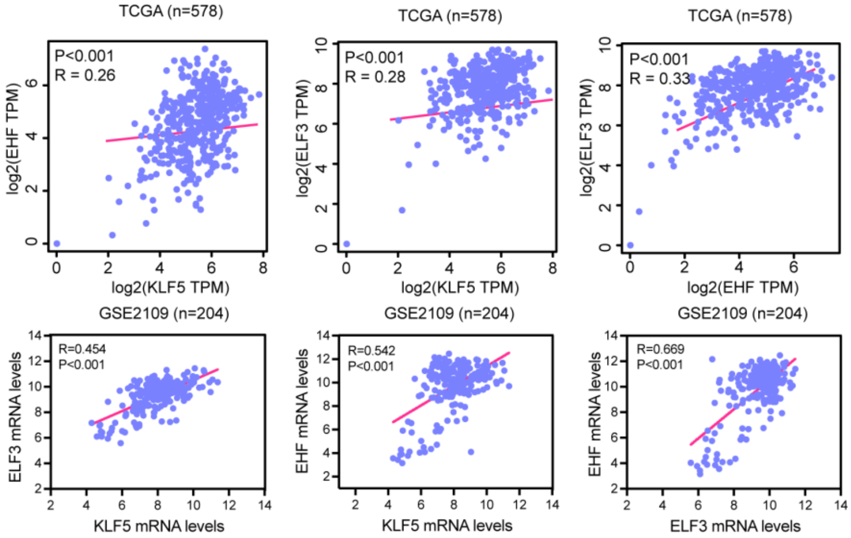

Fig. S7. The expressional correlation between *KLF5*, *EHF* and *ELF3* in OC. (A) The expressional correlation between *KLF5* protein levels and *ELF3* protein levels in FUSSC cohort2. (B) The expressional correlation between *KLF5* protein levels and *EHF* protein levels in FUSSC cohort2. (C) The expressional correlation between *KLF5* mRNA levels, *EHF* mRNA levels and *ELF3* mRNA levels in TCGA cohort and GSE2109 cohort.

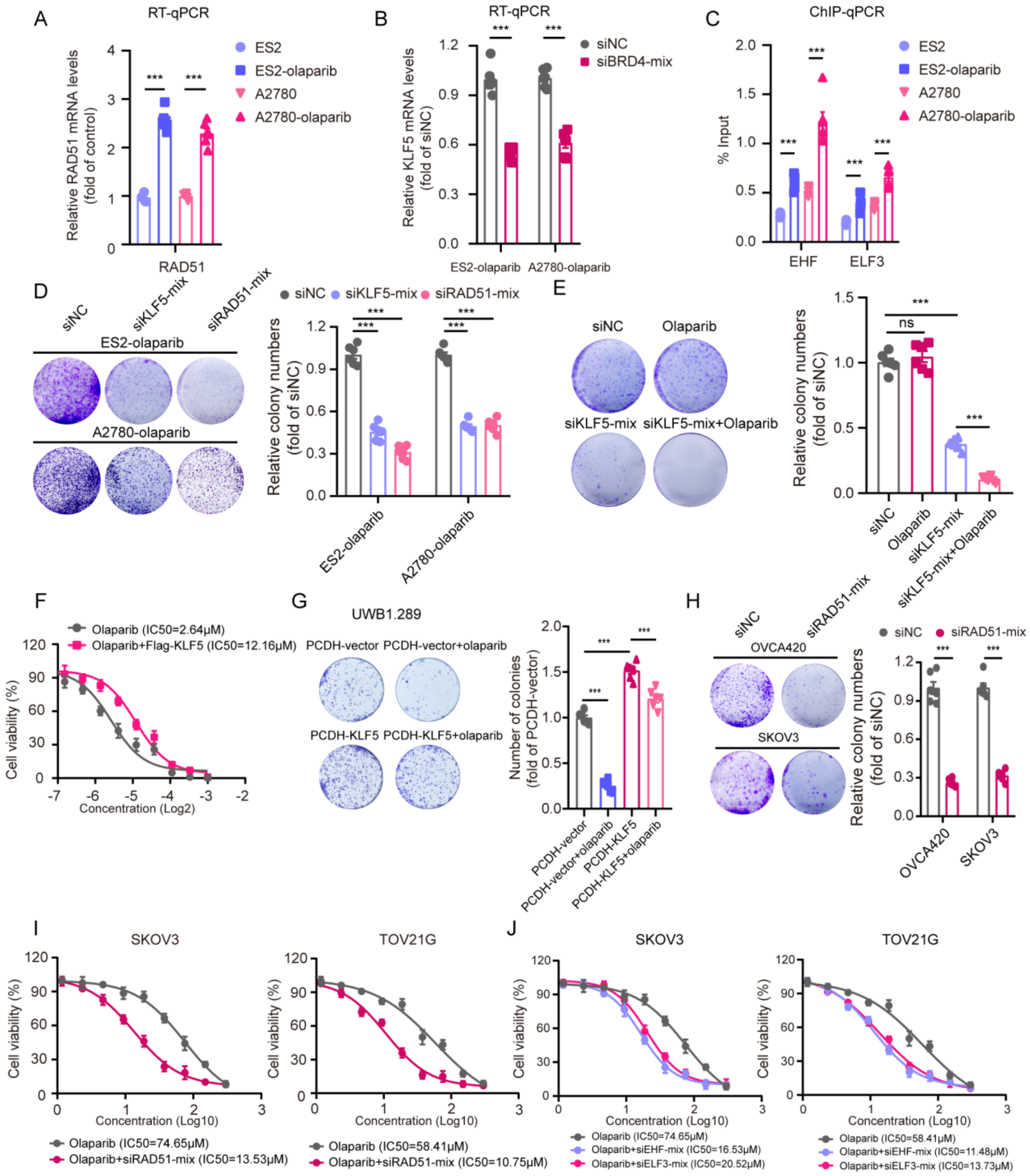

Fig. S8. *KLF5/EHF/ELF3-RAD51* axis regulates PARPi resistance in OC. (A) The *RAD51* mRNA levels in ES2, ES2-olaparib, A2780 and A2780-olaparib cells. (B) The *KLF5* mRNA levels in ES2-olaparib and A2780-olaparib cells transfected with siNC and si*BRD4*-mixed siRNAs. (C) The ChIP-qPCR of *EHF* and *ELF3* in *RAD51* promoter regions in ES2, ES2-olaparib, A2780 and A2780-olaparib cells. (D) Colony formation ability in ES2-olaparib and A2780-olaparib cells transfected with siNC, si*KLF5*-mixed and si*RAD51*-mixed siRNAs. (E) The colony formation ability of SKOV3 cells treated with siNC, olaparib, si*KLF5*-mix RNAs and si*KLF5*-mix RNAs with the combination of olaparib. (F) The IC50 value of olaparib. UWB1.289 OC

cells were infected with PCDH-vector or PCDH-*KLF5* lentiviruses, treated with olaparib and performed CCK-8 assay in 96-well plate. (G) The colony formation ability of UWB1.289 OC cells infected with PCDH-vector or PCDH-*KLF5* lentiviruses and treated with olaparib. (H) The colony formation ability in OVCA420 and SKOV3 cells transfected with siNC or si*RAD51* siRNAs. (I) The IC50 value of olaparib. SKOV3 and TOV21G OC cells were transfected si*RAD51*-mix siRNAs, treated with olaparib and performed CCK-8 assay in 96-well plate. (J) SKOV3 and TOV21G OC cells were transfected si*EHF*-mix and si*ELF3*-mix siRNAs, treated with olaparib and performed CCK-8 assay in 96-well plate. The IC50 value of olaparib was culculated. Values represent the mean ± SEM, n=6. ***P < 0.001.

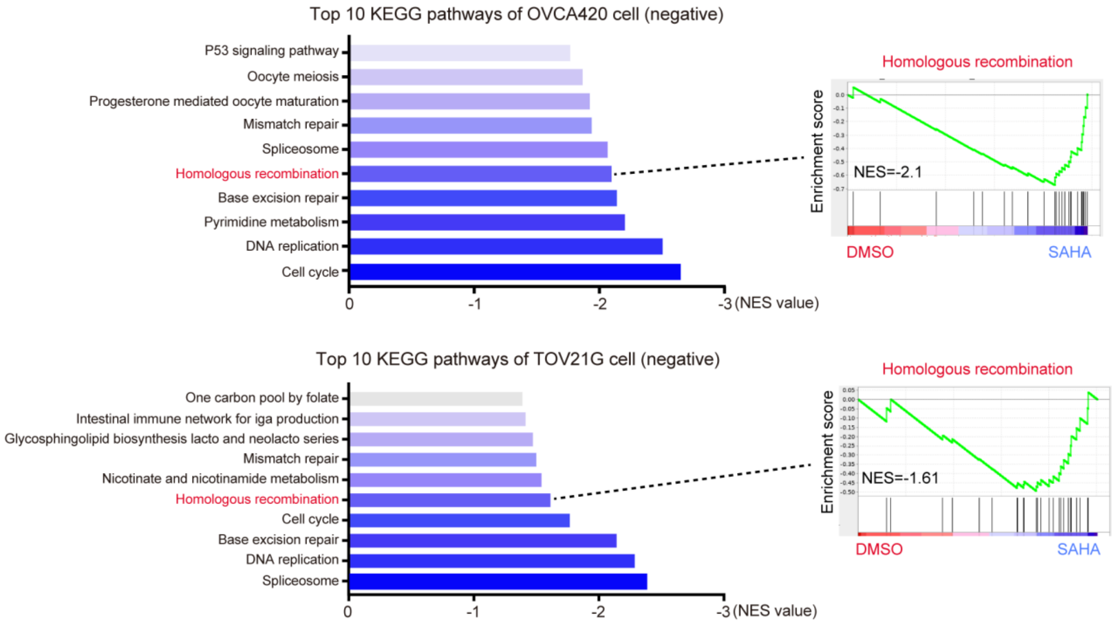

Fig. S9. SAHA regulates HR pathway in OC. OVCA420 and TOV21G ovarian cancer cells were treated with DMSO or SAHA (1 μM, 24h), collected total RNAs and performed RNA-seq. The top 10 negative enriched pathways were analyzed and found the enrichment of homologous recombination pathway in OVCA420 (NES = -2.1) and TOV21G (NES = -1.61) cells.
